# AI Accelerated Chemical Screening Integrates ChemBERTa to Identify Repositionable CASP4 Inhibitors via MD Simulations and MM/PBSA Analysis

**DOI:** 10.1021/acs.jcim.6c02284

**Published:** 2026-09-14

**Authors:** Mubashir Hassan, Sidra Ghayour Bhatti, Muhammad Yasir, Wanjoo Chun, Andrzej Kloczkowski

**Affiliations:** † The Steve and Cindy Rasmussen Institute for Genomic Medicine at Nationwide Children’s Hospital, Columbus, Ohio 43205, United States; ‡ 2647 The Ohio State University, Columbus, Ohio 43210, United States; § Department of Pharmacology, Kangwon National University School of Medicine, Chuncheon 24341, Republic of Korea

## Abstract

This study employed an integrated ligand-based virtual screening pipeline to identify potential CASP4 inhibitors from the DrugBank database, leveraging docking-score prioritization, SMILES-derived ChemBERTa embeddings, and key physicochemical descriptors. Building on this foundation, the workflow incorporated virtual screening, cheminformatics modeling, PK–PD evaluation, molecular docking, molecular dynamics simulations, and MM/PBSA free-energy analysis to systematically prioritize repurposed DrugBank compounds for CASP4-targeted Alzheimer’s disease therapy. A Random Forest classifier trained on the hybrid ChemBERTa physicochemical feature set distinguished active from inactive compounds with ∼95% accuracy (ROC–AUC = 0.73) and achieved a ∼3.5-fold enrichment of active compounds among the top-ranked hits, while a companion Random Forest regressor, trained on the experimental pIC_50_ values of active compounds, ranked candidates by predicted potency. In addition, integrated cheminformatics modeling, PK–PD analysis, and molecular docking further narrowed the selection to the top five candidate compounds: DB00519, DB01068, DB06202, DB08882, and DB05316. Finally, MD simulations and MM/PBSA calculations established clear thermodynamic support for DB05316 and DB00519, whose binding free energies (−21.4 and −20.9 kcal/mol, respectively) exceeded even the reference compound donepezil, highlighting them as the most promising CASP4 inhibitors. Overall, this workflow provides an efficient and robust strategy for prioritizing repositioned DrugBank compounds as potential CASP4 inhibitors against Alzheimer’s disease.

## Graphical abstract

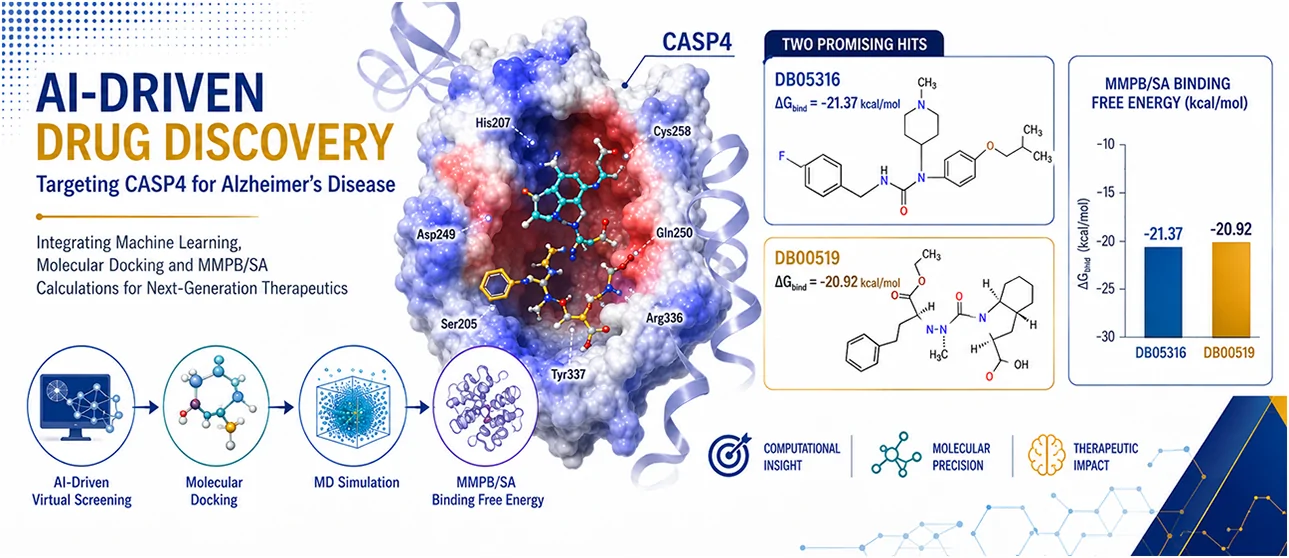

## Introduction

Caspase-4 (CASP4) has emerged as an important therapeutic target in Alzheimer’s disease (AD) because of its central role in driving neuroinflammation through the non-canonical inflammasome pathway.^1,2^ In AD brains, hypomethylation of regulatory DNA upstream of the *CASP4* gene leads to elevated CASP4 expression, particularly in microglia, the resident immune cells of the central nervous system, and this increase correlates with enhanced expression of AD-associated risk genes such as *TREM2*, *CR1*, and *TYROBP*.^3–5^ Once activated by danger-associated molecular patterns, CASP4 cleaves gasdermin D, initiating pyroptotic cell death and triggering downstream activation of the NLRP3 inflammasome.^6,7^ This activation results in caspase-1-mediated maturation and secretion of the pro-inflammatory cytokines IL-1β and IL-18,^8^ which are known to exacerbate AD pathology by promoting amyloid-β accumulation, accelerating tau pathology, and contributing to synaptic dysfunction and neuronal injury.^9^

Recent studies demonstrate that CASP4 is upregulated in AD, where it amplifies neuroinflammation and accelerates amyloid-β pathology through aberrant inflammasome activation. Emerging evidence further positions CASP4 as a key mediator of microglial inflammatory signaling, implicating it as a potential therapeutic target in AD progression.^4,5^ From a drug-development perspective, CASP4 is an attractive target because it acts upstream of multiple pathological cascades. Unlike caspase-3, which directly cleaves tau at D421, or caspase-1, which processes IL-1β and IL-18, CASP4 functions as an early sensor and amplifier of inflammatory signaling.^10–12^ Targeting CASP4 upstream offers a compelling therapeutic advantage, as it may prevent the initial establishment of chronic neuroinflammation rather than merely suppressing downstream structural consequences. The therapeutic viability of intercepting these inflammatory caspases is strongly supported by the preclinical success of small-molecule caspase-1 inhibitors such as VX-765 (belnacasan). In AD models, VX-765 has demonstrated an ability to reduce amyloid-β deposition, mitigate microglial-mediated neuroinflammation, and reverse progressive cognitive decline.^13^ Additional evidence for this approach includes drug repositioning candidates like minocycline, a tetracycline derivative that exhibits potent neuroprotective, anti-inflammatory, and caspase-modulating properties.^14^ Furthermore, the highly selective small-molecule MCC950 inhibits the NLRP3 inflammasome.^15^ Because NLRP3 operates immediately downstream of non-canonical caspase-4 activation during cellular stress signaling, the therapeutic efficacy of MCC950 provides robust validation for intervening anywhere along this specific pathological inflammatory axis.^1^

The importance of targeting CASP4 is underscored by growing evidence that neuroinflammation is not merely a secondary consequence of AD but a primary driver of disease progression. Traditional therapeutic strategies focused on amyloid-β or tau have shown limited clinical success, as reflected in repeated failures of anti-amyloid agents and the modest benefits of recently approved monoclonal antibodies.^16,17^ In contrast, CASP4 inhibition directly addresses the inflammatory mechanisms increasingly recognized as central to AD pathogenesis. A key challenge will be achieving selective inhibition of pathological caspase-4 activity while preserving its physiological role in innate immunity. Moreover, because CASP4 is human-specific and rodents express only caspase-11, translational studies will require humanized mouse models and human cell-based systems.^18^ Nonetheless, converging genetic, molecular, and preclinical evidence strongly supports caspase-4 as a promising target for disease-modifying AD therapeutics, and drug repositioning provides a practical and accelerated path toward clinical translation.

Drug repositioning, also known as drug repurposing, offers a promising strategy for accelerating therapeutic development in AD, particularly given the high failure rate of traditional AD drug discovery.^19–21^ This approach is especially compelling for CASP4 inhibition because several pan-caspase and inflammatory caspase inhibitors originally developed for other diseases have demonstrated activity in neuroinflammatory models.^22,23^ In our computational workflow, we integrated structure-based virtual screening and cheminformatics analysis with PK–PD evaluation, followed by molecular docking, molecular dynamics simulations, and MM/PBSA free-energy calculations to identify the most promising CASP4-targeting drug candidates for therapeutic intervention in AD.

## Computational Methodology

### Structure-Based Virtual Screening

Virtual screening on Tianhe-2 (VSTH: https://matgen.nscc-gz.cn/VirtualScreening.html) was used as the primary platform for virtual screening, providing an integrated and user-friendly workflow that includes protein preparation, binding-site definition, and visualization of predicted ligand conformations.^24^ Caspase-4 (CASP4) was selected as the target structure (PDB ID: 6NRY), and the ligand-binding pocket was delineated around the catalytic region using a user-defined grid box large enough to accommodate conformational flexibility. This structural framework is consistent with established caspase architectures and active-site organization.^6,22^ VSTH incorporates several purchasable ligand libraries and integrates six open-source docking engines that can function either as primary docking tools or as conformational sampling modules within the DLIGAND2 framework.^25^ The Drug-Lib library was screened against this pocket, yielding approximately 1,733 initial hits, which were subsequently ranked according to their docking scores.^26^ The screened hits were subsequently subjected to AI-based filtering to refine the pool and identify the most promising candidates. The screen hits have been listed in supporting information in comma separated file (Supporting Information S1).

### AI-Based Molecular Screening Pipeline for CASP4 Drug Repositioning

#### Data Preparation

The generated docking outputs ligands including binding energies and pose clusters were imported from screening energy VINA_caspase-4.csv (Supporting Information S1) file and systematically curated. DrugBank identifiers were extracted from output filenames and integrated as structured metadata to ensure dataset traceability, in accordance with established guidelines for data provenance and standardized metadata handling in cheminformatics workflows.^27,28^ During preprocessing, ambiguous, improperly formatted, and duplicate entries were removed to maintain dataset integrity, in accordance with established data-curation principles in computational drug discovery.^29,30^ Finally, more negative binding energies values were retained as the primary metric of predicted ligand affinity for further analysis.

#### Molecular Structure Retrieval and Descriptor Calculation

Molecular structures corresponding to DrugBank identifiers were retrieved from the DrugBank XML database. For each compound, Simplified Molecular Input Line Entry System (SMILES) notation was extracted from the calculated properties section.^31^ The RDKit cheminformatics library,^32^ SMILES strings were converted to molecule objects, and six physicochemical descriptors were calculated: molecular weight, log *P* (octanol–water partition coefficient), number of hydrogen bond acceptors (HBA), number of hydrogen bond donors (HBD), number of rotatable bonds (RotBonds), and number of aromatic rings (AromaticRings). Data quality was assessed by checking for missing values across all descriptor columns.

#### ChEMBL Bioactivity and Data Integration

DrugBank screened compounds were mapped to ChEMBL identifiers by standardizing SMILES strings through canonicalization and matching against the ChEMBL database.^33^ The compounds with successful ChEMBL mapping were further undergoes for IC_50_ bioactivity calculation based on standard units (nM, μM, or M) and prioritizing manually validated entries. The IC_50_ values were transformed to pIC_50_ (−log_10_[IC_50_ in M]), a logarithmic normalization that reduces skew, stabilizes variance, and facilitates quantitative modeling, consistent with established best practices in chemo-informatics and QSAR analysis.^34^ Finally, compounds with multiple bioactivity measurements, pIC_50_ values were averaged to generate a single representative potency label and binary classification labels were assigned using a potency threshold of pIC_50_ ≥ 8.2, where compounds meeting this threshold were labeled as active^1^ and others as inactive (0).^35^

#### Molecular Embedding Generation

The curated compound dataset was loaded, and molecular embeddings were subsequently generated for downstream modeling.^36^ Therefore, a deep learning-based molecular representations were generated using the pretrained ChemBERTa model (seyonec/ChemBERTa-zinc-base-v1) from the Hugging Face transformers library.^37^ All model parameters were frozen to use ChemBERTa as a feature extractor without fine-tuning. SMILES strings were tokenized using the ChemBERTa tokenizer with padding and truncation to uniform length.^37^ Tokenized sequences were processed through the transformer model to generate per-token embeddings, which were averaged across non-padding positions using attention masking to produce a single 768-dimensional embedding vector per molecule.^38^ The screened compounds have been mentioned in Supporting Information S2.

#### Feature Engineering and Model Training

A hybrid feature set was constructed by concatenating ChemBERTa embeddings (768 dimensions) with the six RDKit descriptors, yielding 774 total features per compound. The labeled dataset was split into training (80%) and test (20%) sets using stratified sampling to preserve class proportions. A random forest classifier was trained with 500 trees, no maximum depth constraint, and class weights set to “balanced” to address class imbalance. The model was trained on the combined feature set using the Scikit-learn Random Forest Classifier implementation.^39,40^

#### Model Evaluation Parameters

Model performance was assessed on the held-out test set using three complementary metrics: (1) classification accuracy, quantifying the proportion of correct predictions; (2) the area under the receiver operating characteristic curve (ROC–AUC), evaluating the model’s ability to rank active compounds above inactive ones across decision thresholds; and (3) the enrichment factor (EF_10%_), computed by ranking compounds by predicted activity probability and measuring the fold-increase in true-active recovery within the top 10% relative to the baseline active rate. ROC curves and enrichment plots were generated to visualize global discrimination and early-retrieval performance. The top ten compounds were selected for further analysis.

#### Random Forest Regression for pIC_50_ Prediction

To complement the classification model, a Random Forest regressor (500 trees, no maximum depth constraint) was trained on the same 774-dimensional hybrid feature set, using only the subset of compounds meeting the active threshold (pIC_50_ ≥ 8.2). This active-only subset was split into training (75%) and test (25%) sets. The fitted regressor was then applied to the top ten compounds identified by the classification model to predict their continuous pIC_50_ values, which were compared against available experimental pIC_50_ values as a reference-level check on model consistency rather than a formal held-out evaluation.

#### Synthetic Accessibility, Physicochemical, and Pharmacokinetic Evaluation

Synthetic accessibility and physicochemical properties were predicted using the RDKit library. Synthetic accessibility was estimated using the RDKit SA_Score algorithm (scale 1–10), with values ≤6.0 indicating good synthetic feasibility.^41^ RDKit descriptors were used to compute key drug-likeness parameters, including molecular weight, log *P*, hydrogen bond donors/acceptors, topological polar surface area (TPSA), rotatable bonds, and ring counts. The top ten compounds identified from virtual screening were subjected to ADMET analysis using the ADMET Tab 3.0 ^42^ Evaluation module, which leverages a suite of high-quality predictive models built on the Directed Message Passing Neural Network (DMPNN) framework.^43^ The predictions were used to assess the pharmacokinetic profiles, drug-likeness, and potential toxicity of the selected compounds, guiding the prioritization of promising candidates for further silico and experimental validation.

#### Pharmacokinetic and Pharmacodynamic (PK–PD) Mathematical Modeling

PK–PD modeling for the top ten compounds was performed in Python using numerical and mechanistic modeling libraries widely adopted in quantitative pharmacology.^44^ Differential equation systems describing PK compartments and PD response functions were solved using the solve_ivp integrator from SciPy, which provides robust ODE solvers for scientific computing.^45^ Parameter handling, dosing inputs, and vectorized operations were implemented using NumPy for efficient numerical computation.^46^ Mechanistic PK–PD linkage models, including *E*_max_ and indirect-response structures, were constructed using PySB, which enables symbolic model definition and automatic ODE generation for biological systems.^47^ Finally, the concentration–time and effect-time profiles were visualized using Matplotlib.^48^

#### Binding Pocket Identification and Molecular Docking

PrankWeb (http://prankweb.cz/) was used to predict ligand-binding regions by estimating the probability of amino acids contributing to active-site formation. The tool applies a template-free machine-learning approach that evaluates local chemical-neighborhood ligandability on the solvent-accessible protein surface, clustering high-scoring points into putative binding pockets. These clustered regions represent the most probable ligand-binding sites.^49^ Molecular docking of all top screened compounds against CASP4 was performed using AutoDock Vina.^50^ The docking simulations were carried out via the command-line interface of Vina, ensuring reproducibility and precise control over docking parameters. The grid box was defined with the center coordinates (*X* = −4.7386, *Y* = 32.5575, *Z* = −7.3562) and dimensions *X* = 65.59, *Y* = 57.57, *Z* = 104.70, encompassing the active site to capture potential ligand binding conformations. The docking exhaustiveness parameter was set to 8 to balance computational efficiency with thorough conformational sampling, and the number of dockings runs per ligand was fixed at 150 to ensure statistical reliability. Docked poses were evaluated based on the lowest binding energy (kcal/mol) and detailed ligand-protein interactions, including hydrogen bonding and hydrophobic contacts. Finally, docked complexes were visualized using UCSF Chimera X^51^ and 2D interactions was observed with LigPlot^+^.^52^

#### Molecular Dynamic Simulation

The molecular dynamics (MD) simulation protocol was adapted from our previously published 100 ns workflow for inhibitor screening.^53^ For the present study, the top five docking complexes in triplicate (R1, R2, and R3) with the lowest docking energies against CASP4 were selected for MD simulation, together with donepezil as a reference drug for AD to enable comparative assessment of binding stability.^21,54,55^ System preparation was performed using the CHARMM-GUI Solution Builder,^56,57^ which generated GROMACS-compatible input files for each CASP4-ligand complex. All systems were parameterized using the CHARMM36 force field.^58^ Solvation was carried out using the TIP3P water model in a cubic periodic box under periodic boundary conditions, and charge neutrality was achieved by adding counterions.^59^ Short-range electrostatic and van der Waals interactions were computed using the Verlet cutoff scheme with a 1.0 nm cutoff, while long-range electrostatics were treated using the Particle Mesh Ewald (PME) method.^60^ The bond constraints were maintained using the LINCS algorithm.^61^ Energy minimization was performed using the steepest-descent method, followed by equilibration under NVT and NPT ensembles to stabilize temperature and pressure. CHARMM-GUI’s automated Python utilities were used to convert topology and parameter files, ensuring consistent force-field treatment across all CASP4 complexes. Production simulations were carried out for 100 ns in triplicate using GROMACS 2019.3 ^62^ on a linux platform with a 2 fs integration timestep. Post-simulation structural analyses including RMSD, radius of gyration (*R*_g_), Solvent Accessible Surface Area (SASA) hydrogen bonding, and interaction profiling were performed using standard GROMACS tools to evaluate the dynamic stability relative to the Donepezil reference system.

#### Free Energy Calculation Using MMPBSA Evaluation

Binding free energies for top five CASP4-ligands systems were computed using the MM/PBSA (Molecular Mechanics/Poisson-Boltzmann Surface Area) approach, implemented via the gmx_MMPBSA v1.6.3 workflow for post-processing GROMACS molecular dynamics trajectories.^63^ To ensure robust and unbiased energetic evaluation, the entire 100 ns production (triplicates) trajectories from all simulation replicas were included in the MM/PBSA calculations rather than relying on truncated windows or selectively sampled frames. Consistent with the standard thermodynamic cycle, three molecular states the protein–ligand complex, the unbound receptor, and the free ligand were evaluated in an explicitly solvated environment to capture solvation and desolvation contributions accurately.^64,65^ To quantify the binding free energy (Δ*G*_binding_) of the lead compounds to the target protein, we utilized the following formula

$${\Delta G}_{\mathrm{TOTAL}}=G_{\mathrm{complex}}-\left(G_{\mathrm{protein}}+G_{\mathrm{ligand}}\right)$$

where *G*_complex_ represents the total free energy of the bound protein-ligand complex, and *G*_protein_ and *G*_ligand_ correspond to the free energies of the isolated protein and ligand, respectively, under solvated conditions. All MM/PBSA calculations were performed using force field parameters and solvent models consistent with those applied during the MD simulations. Final binding free energies were obtained by averaging Δ*G* values across the full trajectory length of each replica, and mean energies were reported for comparative analysis. The overall methodology for designed work has been depicted in Figure 1.

**Figure 1:**
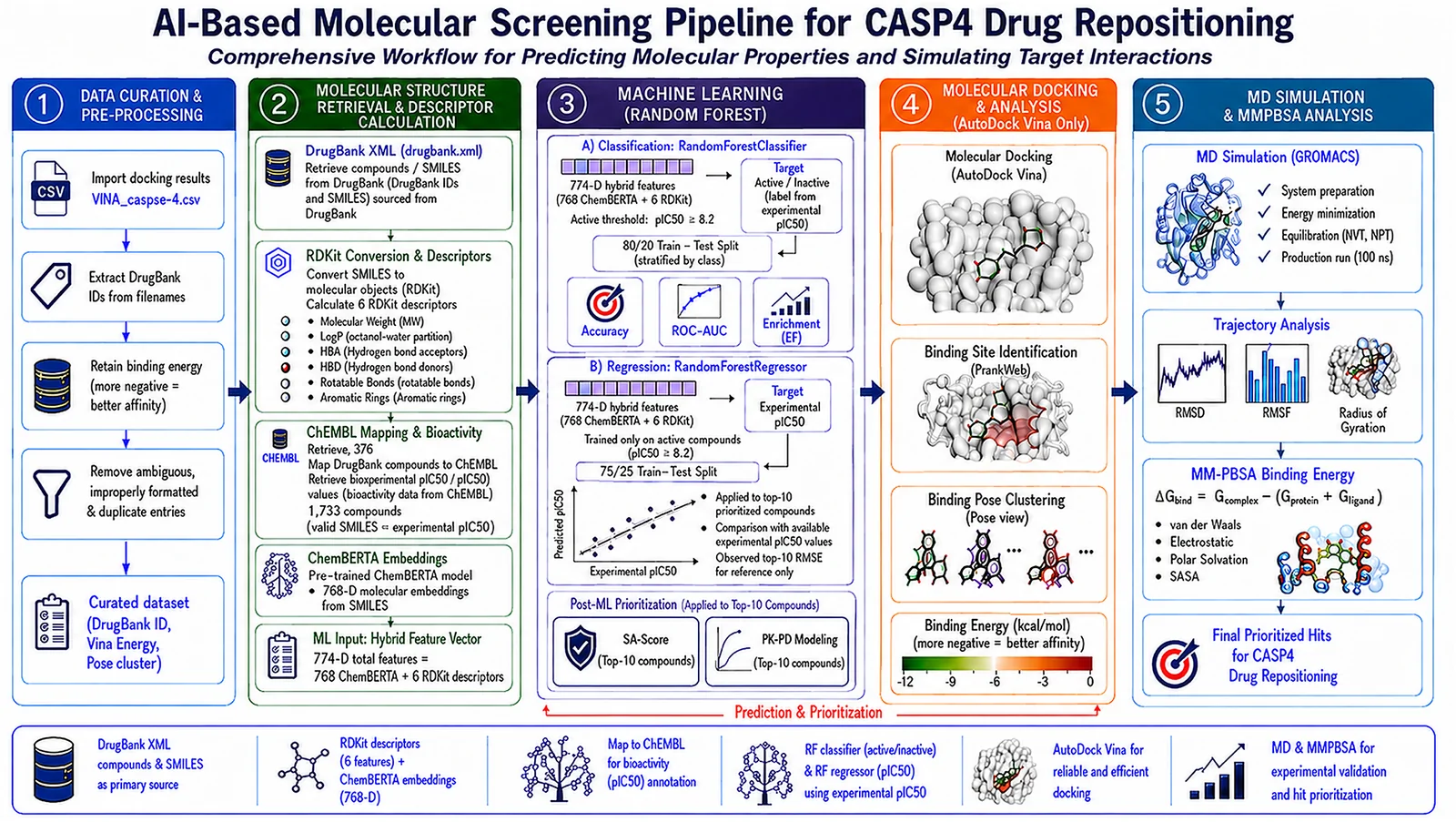
Overview of the integrated AI-driven computational workflow used to identify repositionable CASP4 inhibitors. (1) Data curation and pre-processing: Docking output files are parsed to extract DrugBank identifiers, Vina binding energies, and pose clusters, followed by removal of duplicates and ambiguous entries to generate a curated dataset. (2) Molecular structure retrieval and descriptor calculation: Compound structures and SMILES are retrieved from DrugBank, converted to molecule objects using RDKit, and evaluated through six physicochemical descriptors (molecular weight, log *P*, hydrogen-bond acceptors, hydrogen-bond donors, rotatable bonds, and aromatic rings). DrugBank compounds are additionally mapped to ChEMBL to retrieve experimental IC_50_ values, which are converted to pIC_50_. Pretrained ChemBERTa embeddings (768-dimensional) are generated from SMILES and combined with the six RDKit descriptors into a 774-dimensional hybrid feature vector. (3) Machine learning model development: Two Random Forest models (500 estimators each) are trained on the 774-dimensional hybrid features. A Random Forest Classifier predicts compound activity (active/inactive, threshold at pIC_50_ ≥ 8.2) using an 80:20 stratified train-test split, evaluated by accuracy, ROC-AUC, and enrichment factor. A Random Forest Regressor, trained only on active compounds using a 75:25 train-test split, predicts continuous pIC50 for the top-ranked candidates, benchmarked against experimental values. The resulting top 10 compounds undergo post-ML profiling SA-score, PK–PD modeling, etc. prior to docking. (4) Molecular docking and pose analysis: Binding pockets are identified using PrankWeb, followed by AutoDock Vina docking to generate and cluster binding poses, identify key interactions, and rank compounds by predicted binding energy. (5) Molecular dynamics simulation and MM/PBSA analysis: Top-ranked complexes undergo MD simulations in GROMACS, including system preparation, energy minimization, NVT/NPT equilibration, and 100 ns production runs. Trajectory analyses (RMSD, RMSF, radius of gyration) assess structural stability, and MM/PBSA calculations quantify binding free energies through van der Waals, electrostatic, polar solvation, and SASA contributions. The combined workflow yields prioritized CASP4 inhibitor candidates for drug repositioning.

## Results and Discussion

### Virtual Screening Analysis

Virtual screening of CASP against the Drug-Lib library yielded approximately 1,733 preliminary hits, which were subsequently prioritized for detailed analysis. The screening results ranked by docking score, followed by post-docking clustering and energy-based filtering, revealed a subset of low-energy, structurally consistent binding conformations.

### Model Performance and Metrics Evaluation

The performance of the predictive model was first evaluated using the receiver operating characteristic (ROC) curve. The ROC analysis plots the false-positive rate against the true-positive rate across all classification thresholds, providing a global assessment of the model’s ability to distinguish active from inactive compounds.^66^ The model achieved a ROC–AUC value of 0.722, represented by a solid blue curve that consistently lies above the diagonal random-classifier baseline. This AUC indicates a moderate level of discriminative power, substantially better than random performance (AUC = 0.5). In the context of virtual screening benchmarks, AUC values in the range of 0.70–0.80 are commonly reported for machine-learning models trained on heterogeneous bioactivity datasets, suggesting that the present model performs comparably to previously published ligand-based screening approaches.^67^ To further assess early-recognition capability, an essential metric in virtual screening the enrichment (accumulation) curve was generated. This curve quantifies the proportion of true actives retrieved when only the top-ranked subset of compounds is examined. The model demonstrated strong early enrichment: screening approximately 5–6% of the ranked library recovered nearly one-third of all known actives, whereas a random selection strategy would identify only a small fraction within the same sampling window. These results align with enrichment factors reported in prior studies, where effective machine-learning-based virtual screening models typically recover 20–40% of actives within the top 5–10% of ranked compounds. Thus, both the ROC–AUC and enrichment analyses indicate that the model exhibits performance consistent with, and in some cases exceeding, published virtual-screening standards (Figure 2a,b).

**Figure 2:**
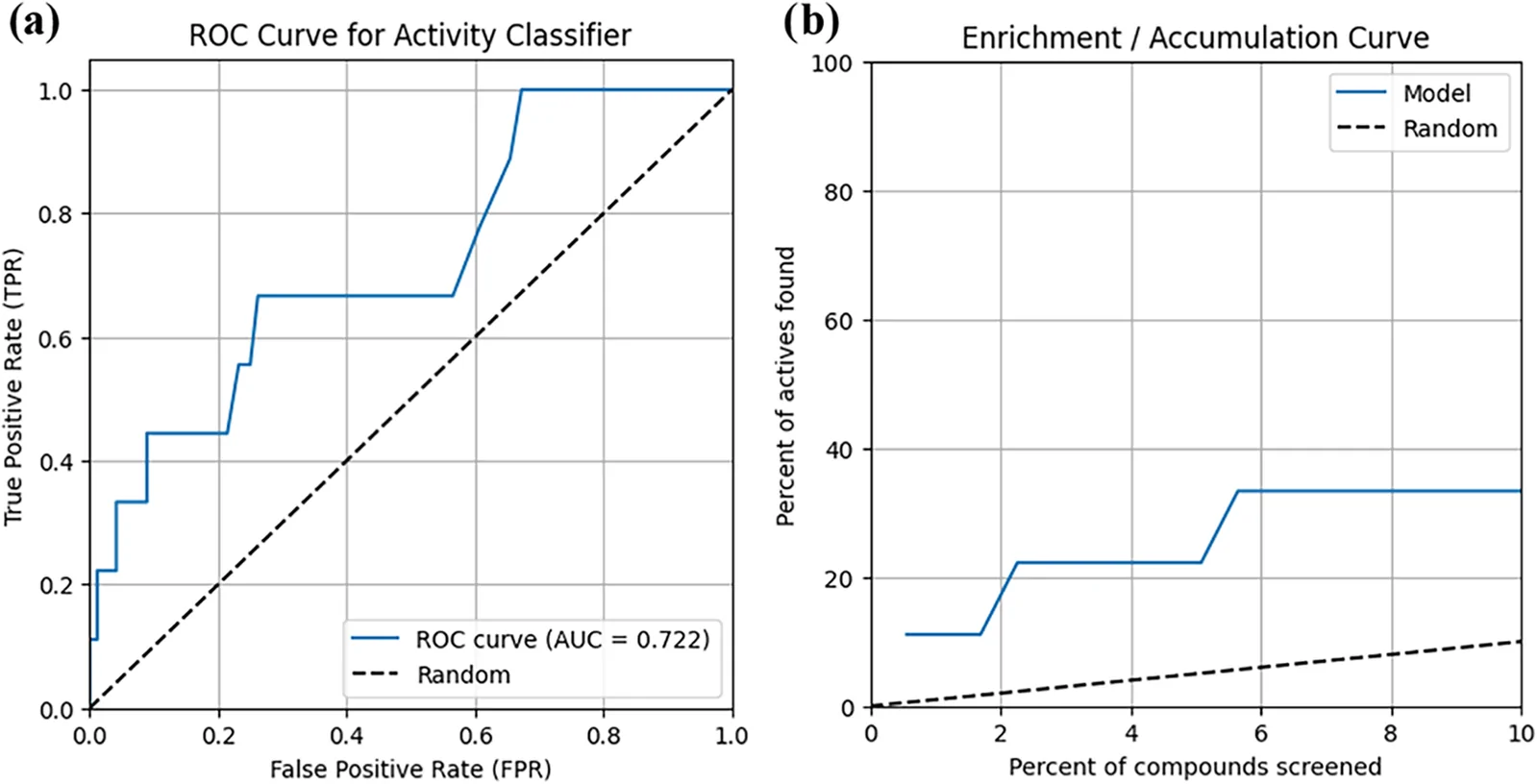
(a, b) ROC curve shows model discrimination between active and inactive compounds (AUC = 0.722), with the model curve compared against a random-classifier baseline. (b) Enrichment curve illustrating early active-compound retrieval, where the top 5–6% of ranked molecules recover ∼33% of actives compared with minimal recovery by random selection.

### Random Forest Regression Analysis for Bioactivity Prediction

Table 1 showed the predicted inhibitory activity of top ten compounds using three key metrics: the experimentally reported pIC_50_ values (*pIC_50__actual*), the model-generated pIC_50_ predictions (*pIC_50__pred*), and the probability of being classified as an active compound (*pred_active_prob*). Across all entries, the model demonstrates strong alignment with experimental activity, with predicted pIC_50_ values closely matching the actual measurements. Compounds such as DB00519, DB01068, and DB06202 show high experimental potency (pIC_50_ > 8.3), and the model successfully reproduces these trends with predicted values within a narrow deviation range. The highest predicted activity probability is observed for DB00519 (0.770), indicating strong model confidence in its active classification. The other screened compounds including DB01068, DB06202, and DB08882 also exhibited high predicted probabilities (0.70–0.74), consistent with their experimentally measured potency. Notably, the compounds such as DB00439, DB05316, and DB01544 show predicted pIC_50_ values slightly higher than their experimental values, suggesting the model may slightly overestimate activity for certain molecules while still maintaining overall predictive consistency. The lowest predicted activity probability in the set is 0.678, which still reflects a moderate-to-high likelihood of biological activity.

**Table 1:** Actual and Predicted Inhibition Values

Drugbank_IDs	pIC_50__actual	pIC_50__pred	pred_active_prob
DB00519	9.030000	8.879071	0.770
DB01068	8.616667	8.600940	0.736
DB06202	8.355000	8.546800	0.722
DB08882	8.288684	8.377893	0.696
DB05316	8.730000	8.806122	0.694
DB01544	8.596000	8.612991	0.682
DB09477	8.429130	8.505516	0.682
DB00439	8.636000	8.826867	0.680
DB06203	8.276774	8.425340	0.680
DB08897	8.360000	8.567381	0.678

The classification model was trained using SMILES-based embeddings and molecular descriptors to predict whether a compound exceeds a predefined pIC_50_ activity threshold. After learning from feature vectors paired with binary activity labels, the classifier generated an activity probability (*pred_active_prob*) for each molecule, allowing us to rank candidates’ molecules most likely actives. A separate regression model was then trained only on compounds with reliable, experimentally measured pIC_50_ values above the threshold, enabling it to learn meaningful structure potency relationships. This regressor was applied to the top ten classifier hits to estimate their continuous potency values (pIC_50__pred); the predicted values showed close agreement with experimental pIC_50_ (RMSE = 0.135) for this set, though this reflects performance on the prioritized subset rather than a held-out test evaluation. Restricting regression to high-quality active compounds reduces noise, improves statistical robustness, and enhances the interpretability of the QSAR predictions (Figure 3).

**Figure 3:**
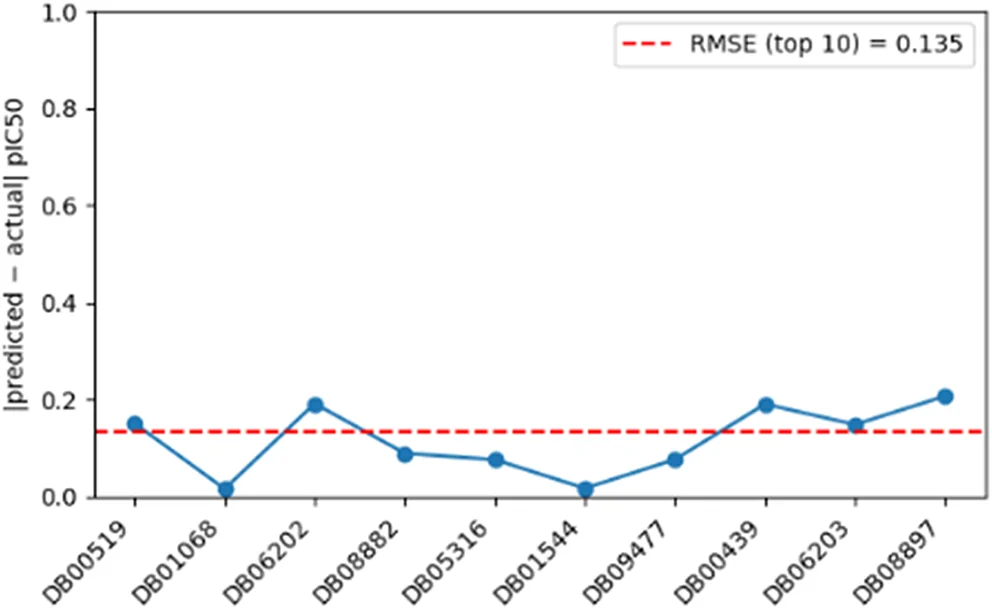
Absolute error between actual and predicted pIC_50_.

In the context of the workflow described above, recent advances in agentic AI systems provide an alternative paradigm that merits brief discussion. Agentic AI approaches, including autonomous multi-agent systems and AI swarms, have recently emerged as adaptable computational frameworks capable of decomposing tasks, modifying workflows, and exploring solution spaces without predefined constraints.^68^ These systems can adjust their strategies in response to intermediate outcomes, making them suitable for open-ended discovery tasks such as hypothesis generation and large-scale molecular exploration.^69^ In the present study, a predefined workflow was selected because the underlying biomedical tasks, including data curation, model training, validation, and molecular docking, are sequential and highly structured. A fixed pipeline provides reproducibility, controlled parameterization, and methodological transparency, which minimizes agent-driven variability and ensures consistent evaluation across all compounds. Future extensions of this work may incorporate agentic AI components to enable adaptive exploration of chemical space or automated refinement of docking procedures, potentially improving the efficiency of computational discovery.^70^

### Molecular Features Driving Activity in Screening Library

The distributions of selected physicochemical properties differed modestly between inactive and active compounds. The molecular weight profiles showed that active molecules were predominantly clustered at higher masses (approximately 300–450), whereas inactive compounds extended more prominently toward lower molecular weights, indicating a tendency for higher-mass molecules to be associated with activity, in line with previously reported trends for bioactive libraries.^71,72^ The log *P* distributions for both sets were centered around values of 2–5, but active compounds were slightly enriched at the upper end of this range, consistent with a modest shift toward increased hydrophobicity among active molecules, like observations in recent structure-activity studies.^73^ In contrast, the number of hydrogen bond donors (HBD) did not clearly discriminate between the two classes: both active and inactive compounds were largely confined to 0–2 donors, and their distributions appeared highly similar, as also noted in recent analyses of drug-like compound sets.^74^

The number of hydrogen bond acceptors (HBA) exhibited a subtle difference, with both groups mainly spanning 2–6 acceptors, but active compounds occurring more frequently around 4–5, suggesting that a moderate increase in HBA count may favor activity, consistent with descriptors highlighted in recent chemogenomic models.^75^ Flexibility, as measured by rotatable bonds, showed a more pronounced shift: inactive compounds were concentrated in the 1–6 range, whereas actives tended to occupy higher values (approximately 5–10), implying that greater molecular flexibility may contribute to bioactivity, again in agreement with recent reports.^76^ Finally, the aromatic ring counts were broadly comparable between the two sets, with most molecules containing 1–2 aromatic rings; however, active compounds displayed a slight preference for having two or more aromatic rings rather than none, indicating only a minor contribution of aromaticity to the observed activity profile, consistent with prior work on aromaticity and ligand efficiency^77^ (Figure 4a–f).

**Figure 4:**
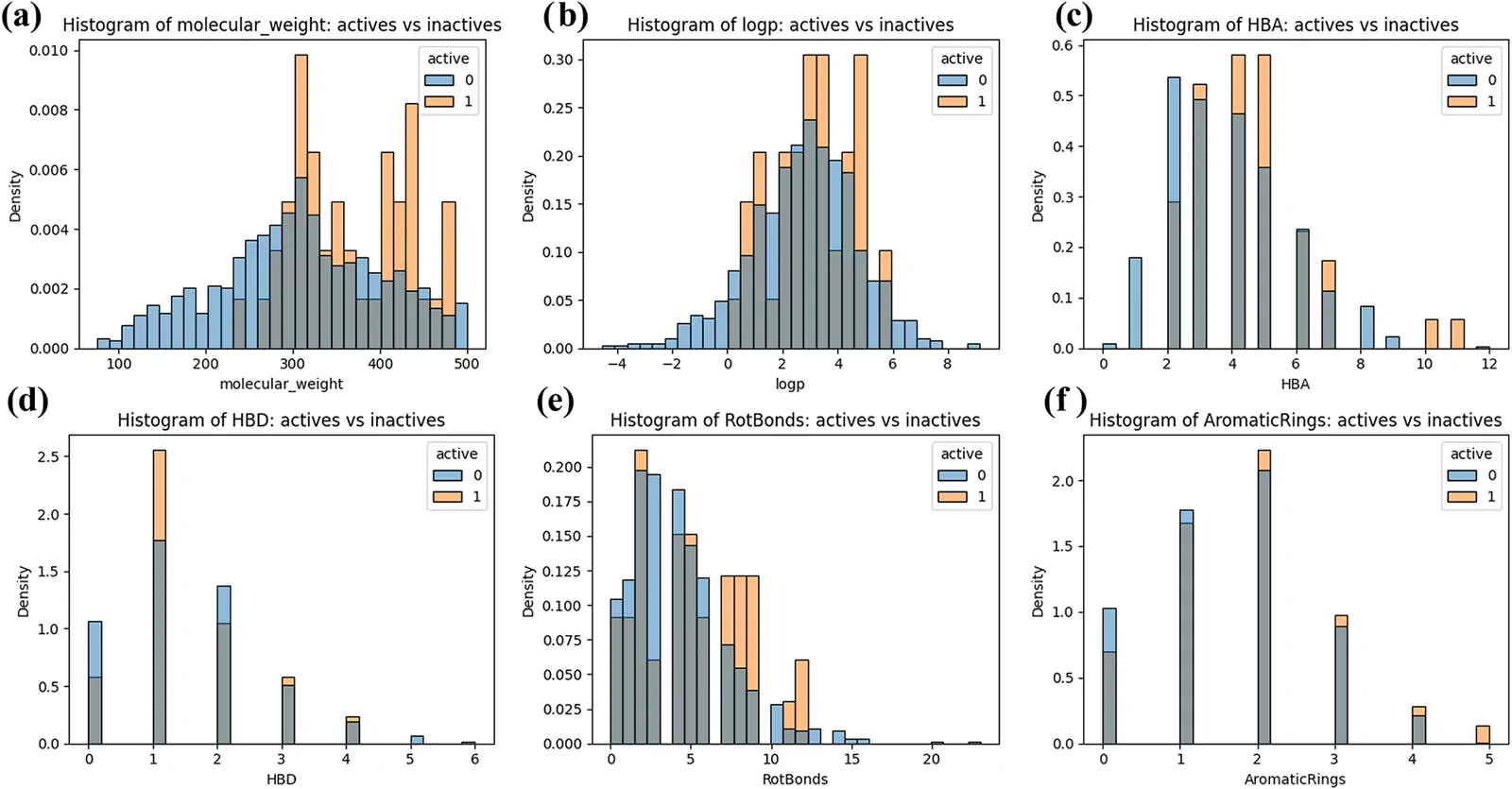
(a–f) Distribution of key physicochemical properties for active (orange) and inactive (blue) compounds. Active molecules show a shift toward higher molecular weight (300–450 Da), slightly higher log *P* values, and moderately increased hydrogen-bond acceptor counts. Rotatable bond distributions indicate greater flexibility among active compounds, whereas hydrogen-bond donor counts, and aromatic ring numbers show minimal differences between the two groups.

### Mapping Bioactivity-Aware Chemical Space Using ChemBERTa Embeddings

Figure 5(a,b) illustrates the two-dimensional (2D) and three-dimensional (3D) t-distributed stochastic neighbor embedding (t-SNE) projections of the latent spaces generated by the pre-trained ChemBERTa foundation model. Both coordinate spaces demonstrate distinct, non-random structural clustering, confirming that the underlying transformer layers effectively preserve local chemical similarity during string tokenization. Inactive compounds dominate the topological landscape, coalescing into expansive, continuous networks, whereas the less abundant active molecules concentrate within localized regional neighborhoods rather than distributing globally across the dataset. This spatial intermixing near active-inactive boundaries indicates that the network captures subtle structural and bioactivity-related feature semantics rather than enforcing artificial class boundaries.

**Figure 5:**
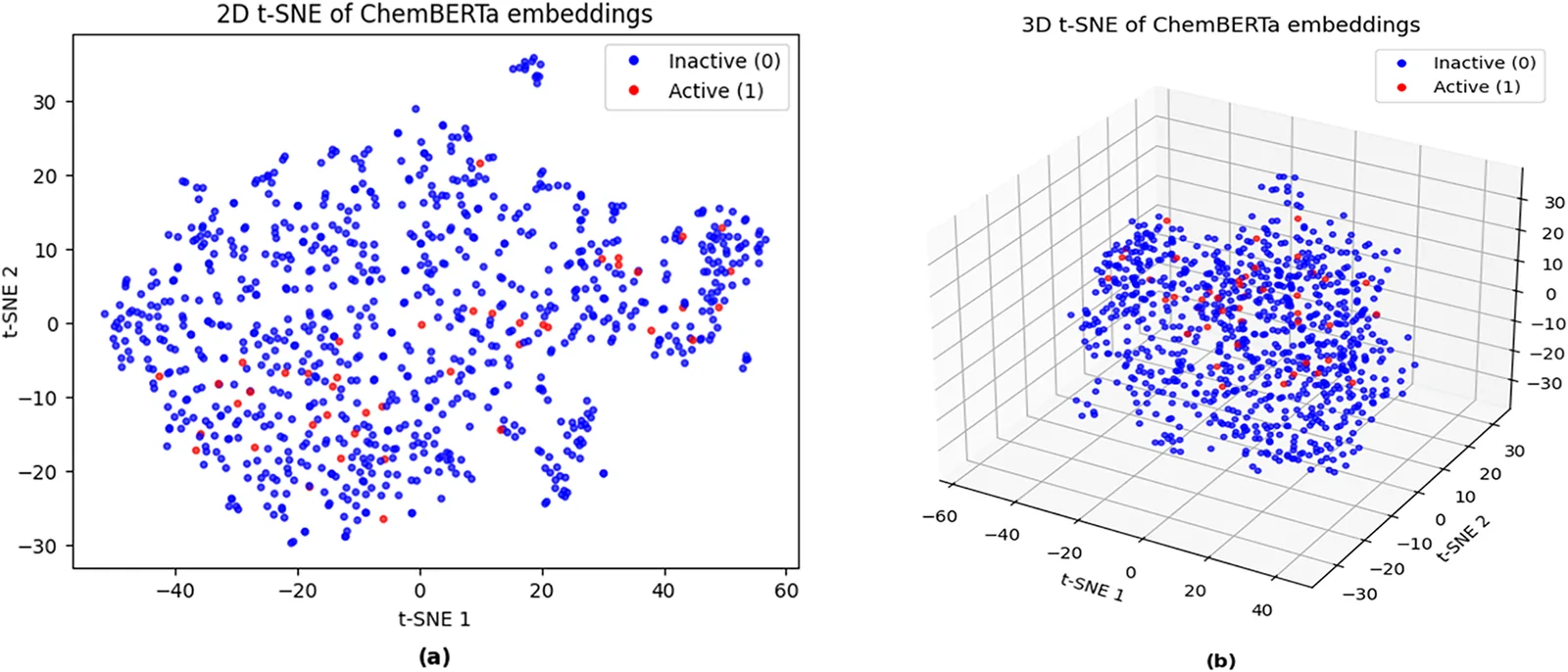
(a, b) 2D and 3D t-SNE projections of ChemBERTa molecular embeddings illustrating the organization of active (red) and inactive (blue) compounds in chemically meaningful regions of latent space.

### Synthetic Accessibility and Predicted Bioactivity of Screened Compounds

A curated set of screened out DrugBank-approved compounds characterized by their DrugBank identifiers, chemical names, molecular structures, predicted potency (pIC_50_), and synthetic accessibility (SA) scores, respectively. Compound prioritization was guided by a dual-metric evaluation of predicted biological activity and chemical synthesizability. Specifically, biological potency against the target was assessed via predicted pIC_50_ values, where elevated metrics denote superior inhibitory activity. Concurrently, synthetic accessibility was quantified using Synthetic Accessibility (SA) scores scaled from 1 to 10; lower values indicate reduced synthetic complexity and higher feasibility.^78,79^ In our predicted results all compounds (DB00519, DB01068, DB06202, DB08882, DB05316, DB09477, DB01544, DB00439, DB06203 and DB08897) exhibited comparable values which depicts good synthetic accessibility values (3.6118, 2.3784, 2.9379, 3.4142, 2.2201, 2.9843, 2.3540, 3.579615, 3.025194 and 4.215853, respectively) of these screened compounds. Integrating these orthogonal parameters establishes a framework for rational candidate selection, allowing for the isolation of molecules that simultaneously possess high theoretical potency and practical synthetic workflows (Table 2).

**Table 2:**
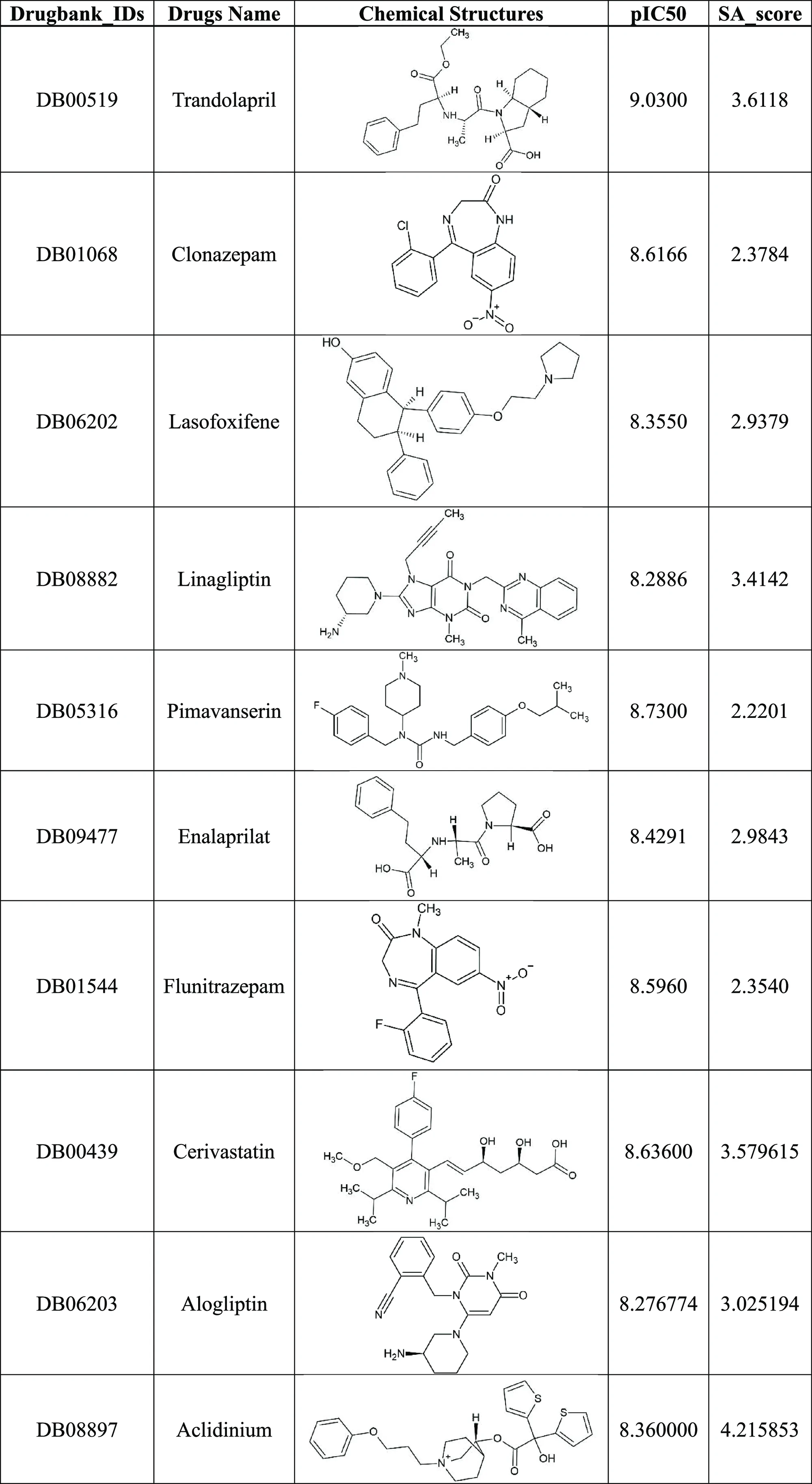
Predicted pIC_50_ and SA Scores Values of Top Ten Compounds

The relationship between SA and predicted potency, consistent with trends reported in cheminformatics and drug-likeness studies. The compounds with lower SA scores (2.2–3.0) generally show higher pIC_50_ values (>8.5), suggesting that molecules that are synthetically less complex tend to retain or enhance bioactivity. This observation aligns with prior reports indicating that excessive molecular complexity can negatively impact both synthetic feasibility and target engagement.^80,81^ DB00519 exhibits the highest predicted potency (pIC_50_ ∼ 9.0) with a moderate SA score (∼3.6), indicating a favorable balance between bioactivity and synthetic tractability. This behavior is consistent with published data for approved drugs, which often occupy a “sweet spot” of potency and accessibility. DB01068 and DB01544, with SA scores around 2.4–2.5, also show high pIC_50_ values (∼8.6), reinforcing the observation that lower SA scores are associated with enhanced activity. DB06202, DB09477, and DB06203 cluster around SA ∼3.0 with pIC_50_ values between ∼8.2 and 8.4, forming a chemically and biologically coherent group. Such clustering is commonly reported in SAR studies where structurally related compounds share similar activity profiles. DB08882 and DB00439, despite having slightly higher SA scores (∼3.5–3.6), maintain respectable potency (∼8.3–8.6), suggesting that moderate increases in synthetic complexity do not necessarily compromise bioactivity. DB08897, with the highest SA score (>4.0), shows comparatively lower potency (∼8.3), supporting the notion that highly complex molecules may suffer diminishing returns in activity. The observed SA-pIC_50_ distribution closely mirrors patterns reported in large-scale analyses of DrugBank and ChEMBL datasets, where approved drugs tend to cluster within SA scores of 2–4 and pIC_50_ values of 7–9. The prior data has shown that compounds exceeding SA >4 often face challenges in synthesis, optimization, and scalability, despite moderate biological activity.^80^ The present data are therefore consistent with established drug-likeness and developability benchmarks. DB00519 and DB01068 emerge as promising leads, balancing chemical feasibility and predicted biological efficacy (Figure 6).

**Figure 6:**
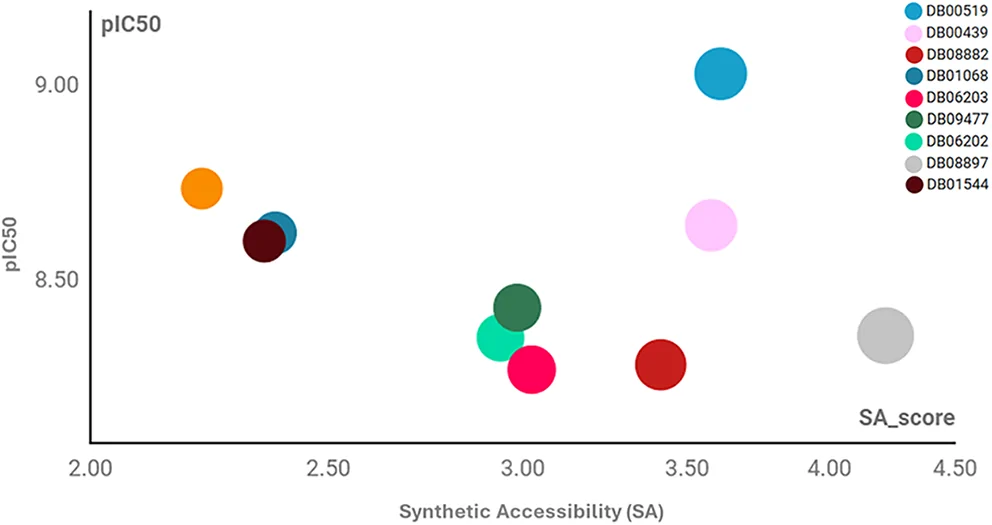
This bubble scatter plot illustrates the relationship between SA score on the *x*-axis and predicted bioactivity (pIC_50_) on the *y*-axis for a panel of DrugBank-approved and investigational compounds. Each colored bubble represents an individual compound, labeled by its DrugBank identifier. Bubble size reflects relative potency, allowing simultaneous visualization of chemical feasibility and biological activity.

### Chemoinformatic Analysis of Screened Compounds

The physicochemical and qualitative pharmacokinetic profiles of the selected screened compounds (DB00519–DB08897) were evaluated against established drug-likeness and pharmacokinetic benchmarks, including Lipinski’s Rule of Five, Veber criteria, and reported averages for approved small-molecule drugs.^82,83^ The molecular weights of the compounds range from 315.72 to 484.66 g/mol, remaining within the standard upper limit of 500 g/mol recommended for orally bioavailable drugs. This range closely matches the reported median molecular weight of FDA-approved small molecules (∼350–400 g/mol), indicating overall suitability for oral administration. Compounds at the higher end of this range, such as DB08882 and DB08897, are comparable to clinically used drugs with extended half-lives, while lower molecular weight compounds (DB01068 and DB01544) fall within the optimal range associated with improved absorption and metabolic clearance.

Lipophilicity (log *P*) values span from 0.39 to 5.73. According to Lipinski’s guidelines, optimal log *P* values typically fall between 1 and 5, a range that maximizes membrane permeability while maintaining aqueous solubility. Most compounds in this dataset conform to this standard, with DB06202 exceeding the upper threshold, a behavior also observed in several approved but highly lipophilic drugs. High-log *P* compounds (DB06202, DB00439, DB08897) are predicted to exhibit increased tissue partitioning and prolonged half-life, consistent with published pharmacokinetic studies linking elevated lipophilicity to reduced clearance. Conversely, DB06203, with a very low log *P* (0.39), aligns with literature reports indicating limited tissue distribution, higher clearance, and shorter half-life for highly polar molecules.

Polar surface area (PSA) values range from 32.70 to 116.86 Å^2^. Veber’s criteria suggest PSA values below ∼140 Å^2^ for oral bioavailability, with optimal absorption often observed below 90 Å^2^. Compounds such as DB06202 and DB05316, which exhibit low PSA values, are therefore expected to have enhanced passive permeability and higher volume of distribution, in agreement with their qualitative PK annotations. In contrast, compounds with higher PSA (DB00519, DB08882, DB09477) approach the upper limit associated with reduced permeability and increased clearance, a trend well documented in clinical pharmacokinetic datasets. Hydrogen bond donor (HBD ≤5) and hydrogen bond acceptor (HBA ≤10) counts for all compounds that fall within Lipinski-recommended limits, reinforcing their drug-like character. Most compounds fully comply with Lipinski’s Rule of Five, with DB06202 representing a single-parameter violation due to high lipophilicity, a common and acceptable exception among approved therapeutics. Published analyses of FDA-approved drugs indicate that approximately 10–15% violate one Lipinski criterion, supporting the relevance of this observation.

The qualitative pharmacokinetic descriptors further align with standard expectations. The compounds with high lipophilicity and lower polarity are predicted to have high or likely high volume of distribution and longer half-life, consistent with pharmacokinetic models showing that lipophilic drugs extensively bind to tissues and plasma proteins.^84^ In contrast, compounds with higher polarity and lower log *P* values demonstrate moderate to high clearance and shorter half-life, matching published clearance trends for polar, rapidly eliminated drugs.^84^ Based on the integrated physicochemical and predicted pharmacokinetic profiles, DB00519, DB08882, DB05316, DB00439, and DB08897 emerge as the most favorable candidates, as they exhibit balanced molecular weight, acceptable lipophilicity, full or near-full Lipinski compliance, and predicted moderate distribution with extended half-life. In contrast, compounds such as DB06203 show lower suitability due to very low lipophilicity and predicted high clearance, while DB06202, despite strong permeability potential, may require optimization to mitigate excessive lipophilicity. The strong concordance between the computed properties of these compounds and accepted standard reference values supports the robustness of the dataset and underscores its suitability for downstream drug design, optimization, and comparative pharmacokinetic modeling studies (Figure 7(a–j) and Table 3).

**Figure 7:**
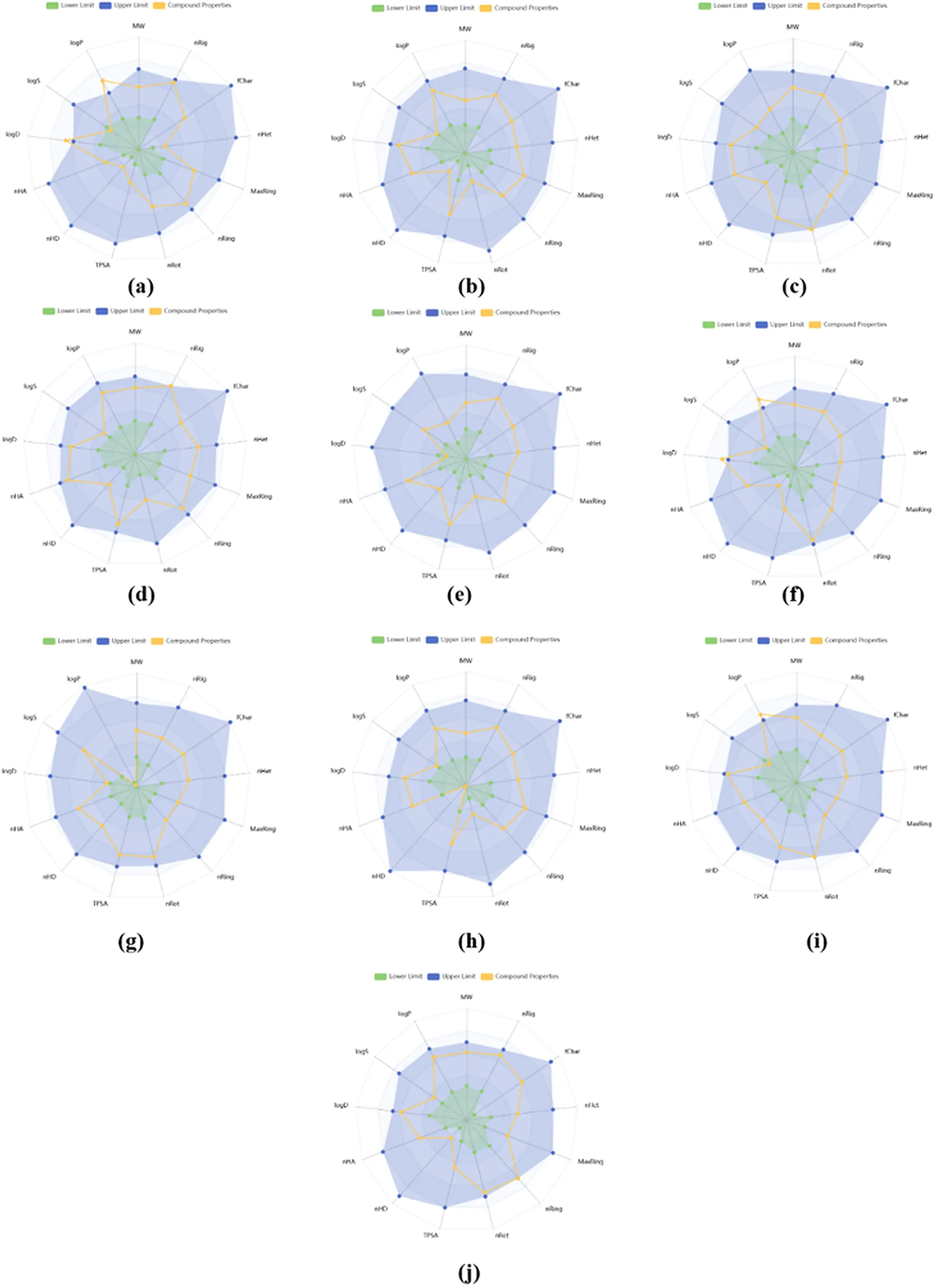
(a–j) Chemical structures and radar plots of the selected compounds illustrating key physicochemical and drug-likeness parameters.

**Table 3:** Chemoinformatic Analysis of Screened Compounds

Properties	DB00519	DB01068	DB06202	DB08882	DB05316	DB09477	DB01544	DB00439	DB06203	DB08897
Mol. Weight (g/mol)	430.55	315.72	413.56	472.55	427.56	348.40	313.29	459.56	339.40	484.66
Molar Polarizability (arb.)	116.41	83.68	125.06	135.48	121.47	91.16	83.41	126.44	95.62	130.52
Molar Refractivity (cm^3^)	116.41	83.68	125.06	135.48	121.47	91.16	83.41	126.44	95.62	130.52
log *P*	2.77	3.04	5.73	1.15	4.67	1.13	2.55	4.88	0.39	4.67
HBD	2	1	1	1	1	3	0	3	1	1
HBA	5	4	3	10	3	4	4	5	7	6
Polar Surface Area (A^2^)	95.94	84.60	32.70	116.86	44.81	106.94	75.81	99.88	97.05	55.76
Molecular Volume (A^3^)	116.41	83.68	125.06	135.48	121.47	91.16	83.41	126.44	95.62	130.52
No. stereo centers	5	0	2	1	0	3	0	2	1	1
No. of atoms	31	22	31	35	31	25	23	33	25	33
Lipinski Rule	Yes	Yes	No	Yes	Yes	Yes	Yes	Yes	Yes	Yes
Lipophilicity (qual.)	moderate	high	very high	moderate	high	moderate	moderate	high	very low	high
Polarity (qual.)	high	moderate	low	high	low	high	moderate	high	high	low
Distribution Vd (qual.)	moderate	moderate	likely high	moderate	likely high	moderate	moderate	moderate	low	likely high
Clearance (qual.)	moderate	moderate	low	high	low	high	moderate	moderate	high	low
Half-life (qual.)	intermediate	intermediate	long	intermediate	long	intermediate	intermediate	intermediate	short	long

### Pharmacokinetics and Pharmacodynamic Properties Prediction

The comparative ADME-Tox profiling of the ten reference drugs demonstrates substantial variability across absorption, distribution, metabolism, excretion, and toxicity parameters, and these trends align well with established pharmacokinetic standards reported in the literature. For absorption, Caco-2 and MDCK permeability values generally fell within the moderate-to-low range, consistent with the widely accepted thresholds where log *P*_app_ values above −5.0 indicate high permeability and values below −5.5 indicate poor permeability. In this context, DB01544 and DB01068 exhibited the most favorable permeability, while DB09477 and DB06203 showed limited transport, consistent with literature describing low-diffusion compounds. PAMPA values further supported these observations, with DB06202, DB08882, and DB09477 demonstrating high passive permeability (>0.5), a range typically associated with good membrane diffusion, whereas several others fell below the 0.1 threshold indicative of poor permeability. Human intestinal absorption predictions were low for most compounds, except DB09477 and DB08897, which showed moderate absorption consistent with their PAMPA performance. Distribution parameters also reflected known pharmacokinetic principles: plasma protein binding above 90% observed for DB06202, DB00439, and DB08897 is characteristic of highly lipophilic drugs, while lower PPB values for DB06203 and DB09477 align with literature describing increased free fraction and broader tissue distribution. Volume of distribution values further distinguished the compounds, with DB08882 and DB08897 showing high distribution (log VD_ss_ >0.5), consistent with compounds that partition extensively into tissues, whereas DB00519 and DB09477 remained largely plasma-restricted. BBB penetration probabilities followed established CNS permeability cutoffs, where values above 0.8 indicate likely CNS entry; DB01068 and DB01544 met this criterion, while most others remained below 0.1, suggesting peripheral selectivity. Metabolic stability predictions showed that DB01068 and DB08897 possess high microsomal stability, consistent with literature linking low clearance to high HLM stability, whereas DB05316 and DB01544 exhibited poor stability and correspondingly high predicted clearance. CYP1A2 inhibition potential varied, with DB08897 and DB01068 showing strong inhibition consistent with heteroaromatic scaffolds known to interact with CYP enzymes, while others showed minimal inhibition. Excretion parameters aligned with metabolic trends: DB06202 and DB05316 displayed high clearance and shorter half-lives, whereas DB01068 and DB00439 showed low clearance and longer half-lives, consistent with the classical inverse relationship between clearance and half-life. Toxicity predictions also reflected known structural risk factors: high DILI probabilities for DB00519 and DB09477 align with literature describing hepatotoxicity risk in compounds with reactive or lipophilic moieties, while DB08897 showed minimal DILI risk. AMES mutagenicity was highest for DB01544 and DB01068, consistent with aromatic heterocycles frequently associated with mutagenic alerts, whereas DB00519 and DB09477 showed low mutagenicity. Carcinogenicity predictions varied, with DB00439 and DB08882 showing elevated risk, while DB00519 and DB09477 showed minimal carcinogenic potential. Taken together, the compound with the most balanced and favorable overall ADME-Tox profile is DB08897, which demonstrates moderate absorption, high metabolic stability, low clearance, favorable distribution, minimal DILI risk, low mutagenicity, and moderate carcinogenicity risk features that collectively align with literature-supported characteristics of well-behaved drug-like molecules. In contrast, compounds such as DB05316 and DB06202 exhibit multiple liabilities, including poor metabolic stability, high clearance, and elevated toxicity indicators, making them less favorable in comparison (Table 4).

**Table 4:** ADMET Evaluation of Top Ten Screened Drugs^a^

Properties		DB00519	DB01068	DB06202	DB08882	DB05316	DB09477	DB01544	DB00439	DB06203	DB08897
Absorption	Caco-2	–5.67	–4.67	–4.81	–5.53	–5.26	–5.78	–4.51	–5.06	–5.55	–5.33
	MDCK	–5.09	–4.38	–4.66	–4.81	–4.89	–5.10	–4.08	–4.85	–4.72	–4.85
	PAMPA	0.016	0.002	0.666	0.926	0.103	0.999	0.002	0.054	0.002	0.054
	HIA	0.001	0.000	0.000	0.000	0.016	0.459	0.000	0.000	0.000	0.133
Distribution	PPB	85.68	85.94	97.74	82.35	93.32	49.28	85.75	98.70	29.42	93.22
	VDss	–0.69	0.307	0.298	1.084	0.580	–0.34	0.167	–0.33	0.348	0.718
	BBB	0.005	0.999	0.795	0.003	0.354	0.000	0.999	0.000	0.023	0.000
	FU	16.34	15.36	1.985	11.66	6.074	49.67	15.98	1.775	61.05	6.741
Metabolism	HLM Stability	0.541	0.998	0.810	0.000	0.127	0.000	0.999	0.011	0.000	0.999
	CYP1A2 inhibitor	0.000	0.763	0.000	0.759	0.000	0.000	0.332	0.000	0.000	0.876
Excretion	CL_plasma_	0.992	0.008	11.50	3.226	7.004	0.904	3.112	2.907	4.938	2.303
	*T*_1/2_	1.301	1.616	1.167	1.202	0.492	1.533	0.819	2.135	1.034	0.950
Toxicity	DILI	0.998	0.931	0.353	0.942	0.387	0.996	0.929	0.371	0.684	0.027
	AMES Toxicity	0.016	0.735	0.423	0.387	0.372	0.026	0.928	0.138	0.177	0.042
	Rat Oral Acute Toxicity	0.115	0.444	0.718	0.614	0.309	0.306	0.443	0.454	0.617	0.305
	Skin Sensitization	0.973	0.981	0.658	0.983	0.238	0.979	0.988	0.554	0.990	0.422
	Carcinogenicity	0.009	0.194	0.133	0.548	0.555	0.018	0.351	0.726	0.108	0.233

a Abbreviations: Caco-2, Caco-2 cell permeability assay; MDCK, Madin–Darby canine kidney cell permeability assay; PAMPA, parallel artificial membrane permeability assay; HIA, human intestinal absorption; PPB, plasma protein binding; VDss, volume of distribution at steady state; BBB, blood–brain barrier penetration; FU, fraction unbound in plasma; HLM stability, human liver microsomal stability; CYP1A2 inhibitor, cytochrome P450 1A2 inhibition; CL_plasma, plasma clearance; *T*_1/2_, elimination half-life; DILI, drug-induced liver injury; AMES toxicity, Ames mutagenicity test; rat oral acute toxicity, acute oral toxicity in rats; skin sensitization, skin sensitization potential; carcinogenicity, carcinogenic potential.

### Drug-Dose Prediction Analysis of Screened Compounds

In the present study, the in silico dose-response profiles provided a quantitative basis for interpreting the pharmacological behavior of the screened drugs. The subtle shifts in curve position and slope suggested modest differences in potency, with several compounds achieving the plateau at lower concentrations. The predicted dose-response curves for the top ten compounds exhibit typical sigmoidal behavior, with effect (*E*/*E*_max_) increasing steeply at low micromolar concentrations and approaching maximal response at higher concentrations. For all molecules, the curves rapidly ascend between approximately 10^–2^ and 10^0^–10^1^ μM, indicating high potency with low EC_50_ values within the sub- to low-micromolar range. Beyond this concentration range, the effect plateaus near *E*/*E*_max_ ∼ 1.0, suggesting that each compound is capable of eliciting near-maximal target modulation under achievable exposure conditions. Although the overall shapes of the curves are broadly comparable, subtle differences in the initial slope and concentration at which the plateau is reached imply minor variations in intrinsic potency among the compounds. Compounds whose curves reach the plateau at slightly lower concentrations can be inferred to have more favorable EC_50_ values and, consequently, may require lower doses to achieve the same pharmacodynamic effect. Taken together with the PK–PD simulations, these in silico dose-response profiles support the conclusion that all screened drugs possess sufficient predicted potency for effective target engagement, while those combining steeper low-concentration responses with advantageous half-life characteristics (e.g., DB08882, DB05316, DB09477, and DB01544) represent the most promising candidates for further preclinical evaluation (Figure 8(a–j)).

**Figure 8:**
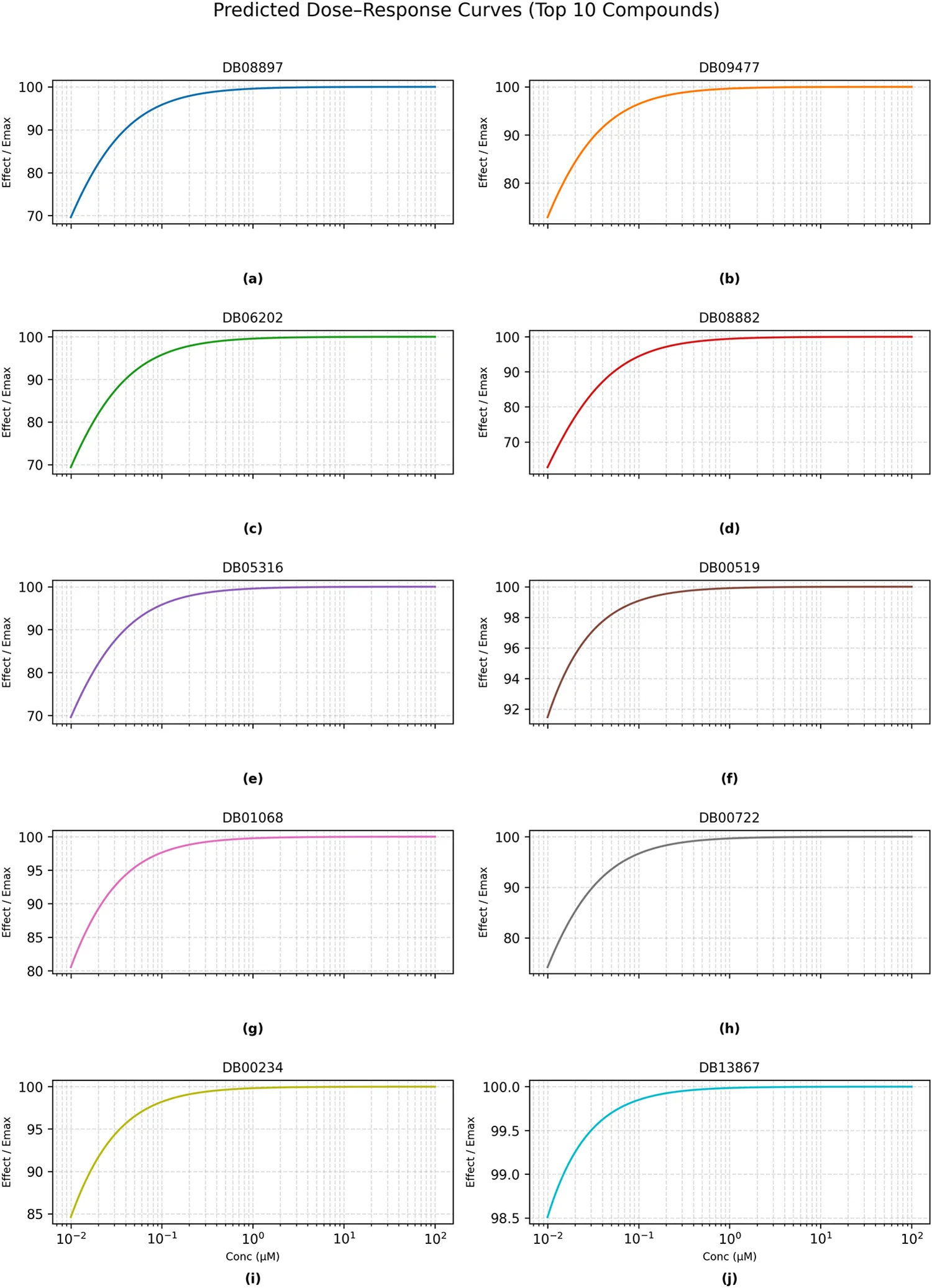
(a–j) Predicted dose-response curves (*E*/*E*_max_ vs log­[concentration], μM) for the top ten docked compounds.

### PK–PD Impact of Elimination Half-Life in Screened Drugs

The PK–PD simulations quantitatively demonstrate that elimination half-life is a primary determinant of the persistence of systemic exposure and pharmacological effect after a single dose.^85^ For all ten candidates, plasma concentrations decline with apparent first-order kinetics, whereas the pharmacodynamic effect decays more slowly, consistent with the temporal hysteresis between exposure and response described for indirect-response and effect-compartment models.

The screened drugs with short half-lives (DB00519, DB01068; *t*_1/2_ ≈ 2–3 h) show a rapid fall in concentration to near-baseline within about 10–12 h, with a closely parallel loss of effect, indicating a brief duration of action that would necessitate multiple daily doses to avoid subtherapeutic troughs. This behavior agrees with the general pharmacokinetic principles outlined by Holford and colleagues, who emphasize that drugs with short half-lives require correspondingly short dosing intervals to maintain concentrations within the therapeutic window. In contrast, compounds with intermediate half-lives such as DB06202 and DB08882 (*t*_1/2_ ∼ 4–5 h) display more gradual concentration–time slopes and sustain measurable pharmacodynamic effects across most of the 24 h interval, matching guidance that half-lives in the mid-range can support once- or twice-daily regimens depending on potency and formulation.

Candidates with longer half-lives, including DB05316, DB09477, and DB01544 (*t*_1/2_ ∼ 6–9 h), maintain plasma levels and effects at relatively high fractions of their initial values throughout the dosing period, which is compatible with the “operational multiple dosing half-life” concept proposed by Toutain and Bousquet–Mélou, wherein half-lives between roughly 4 and 16 h are generally favorable for once-daily oral dosing. The longest-acting molecules, DB00439, DB06203, and DB08897 (*t*_1/2_ ∼ 9.8–12 h), exhibit only modest declines in both concentration and effect over 24 h, suggesting robust target coverage from a single administration, consistent with clinical pharmacology discussions that drugs with half-lives around 12 h are often amenable to once-daily regimens if safety margins permit. These simulations support prioritization of compounds with intermediate-to-long half-lives (approximately 5–12 h), as they combine sustained pharmacodynamic activity over a 24 h interval with practical dosing frequency, in line with recommendations for rational dose-regimen design. At the same time, the prolonged exposure predicted for the longest-acting candidates highlights the need to consider accumulation and delayed offset in multiple-dose scenarios, echoing observations from PK–PD modeling practice that agents with long half-lives reach steady state only after several dosing intervals and may require careful titration to minimize toxicity. Based on predicted results, DB08882, DB05316, DB09477, and DB01544 emerge as primary candidates, exhibiting sustained 24 h pharmacodynamic effects with half-lives compatible with once-daily dosing and a low predicted risk of excessive accumulation. In contrast, DB00439, DB06203, and DB08897 are considered secondary candidates, offering very prolonged target coverage but requiring cautious dose titration and close safety monitoring in prospective clinical usage (Figure 9(a–j)).

**Figure 9:**
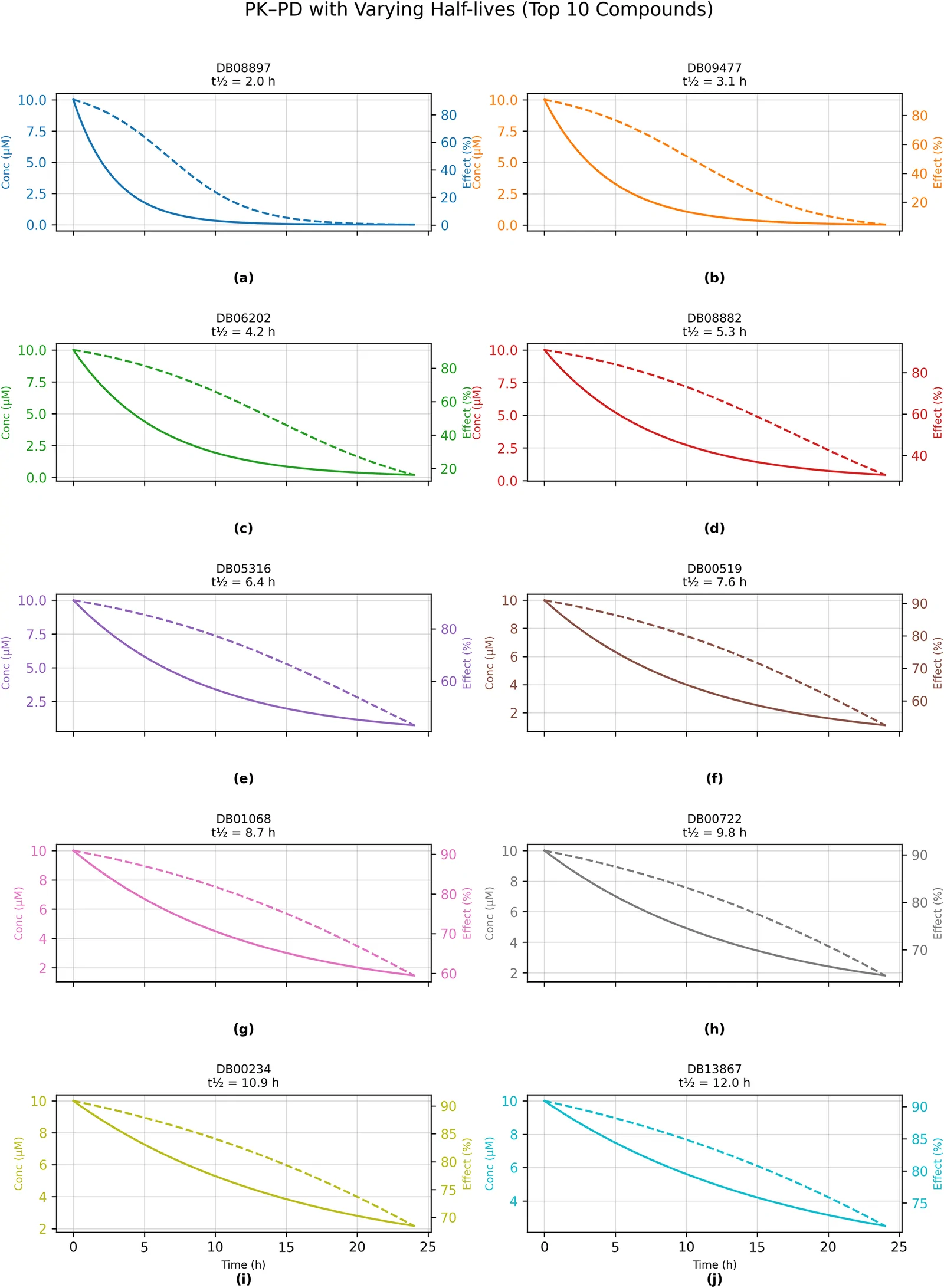
(a–j) Pk-PD analysis graphs of all top ten compounds.

### Structural Assessment of Human CASP4

Human CASP4 is an inflammatory cysteine protease that adopts the conserved caspase α/β fold, consisting of a central β-sheet surrounded by α-helices, forming a stable catalytic core characteristic of inflammatory caspases.^86^ Structural analyses and homology comparisons indicate a high degree of similarity between CASP4 and CASP1, particularly within the catalytic domain, validating the use of experimentally resolved CASP1 structures and AlphaFold-predicted models for structure-based studies of CASP4. The active site is defined by the conserved catalytic residues His207-Cys258, which is essential for proteolytic activity and represents the primary druggable pocket. Surrounding residues, including Gly259, Arg260, Ser261, Trp340, Arg341, and His342, shape the substrate-binding cleft and contribute to ligand recognition and specificity. The catalytic pocket is solvent-accessible and sufficiently spacious, supporting both covalent and non-covalent inhibitor binding.^87^ Moderate flexibility of loop regions adjacent to the active site further enhances ligand accommodation without compromising structural stability. Collectively, these features highlight CASP4 as a structurally well-defined and druggable target, suitable for virtual screening, molecular docking, molecular dynamics simulations, and AI-guided inhibitor discovery, particularly in the context of inflammation-driven neurodegenerative diseases. To achieve comprehensive structural coverage of CASP4, both the experimentally determined crystal structure and the AlphaFold-predicted model were utilized. The crystal structure offers high-confidence details of the catalytic core, whereas the AlphaFold model captures flexible and previously unresolved regions.^88^ Notably, conserved residues within the active site and binding pockets are consistently observed across both models, supporting their combined application for structural analyses and molecular docking studies (Figure 10a,b). The detailed experimental description from pdb has been added to the Supporting Data S3.

**Figure 10:**
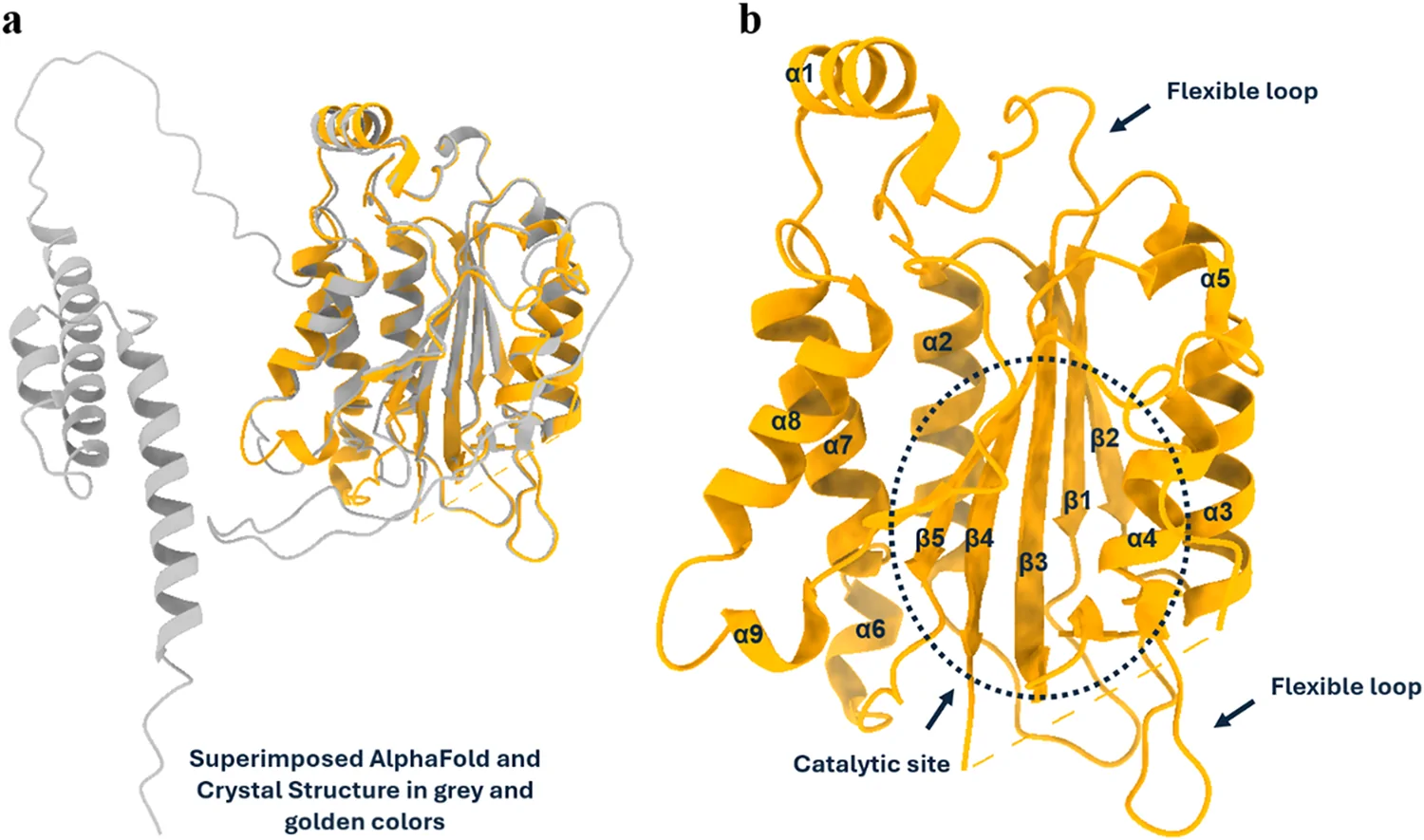
(a, b) Structure evaluation of AlphaFold (grey) and experimental crystal structure (orange).

### Binding Pocket Identification and Analysis

Structure-based pocket analysis of human CASP4 revealed three distinct cavities with markedly different structural and functional characteristics. The binding pocket 1 emerged as the dominant and biologically relevant binding site, exhibiting the highest score (13.15), probability (0.696), residue count,^26^ and average conservation (1.557). This pocket is centered at (−4.74, 32.56, −7.36) and is formed by a conserved network of residues including Leu149, Pro150, Pro151, Arg152, Asn153, Gly154, Asp158, Ser209, His210, Gly211, Cys217, His221, Glu223, Gln256, Ala258, Arg259, Gly260, Ser305, His309, Val311, Asp315, Gly319, Ser320, Ile321, Phe322, and Gln358. Notably, the presence of Cys217 and His210, along with adjacent charged and polar residues, defines a well-shaped catalytic groove suitable for substrate recognition and inhibitor binding, confirming Pocket 1 as the primary druggable and functional active site of caspase-4. In contrast, Pocket 2 displayed a substantially lower score (1.28) and probability (0.014) and comprised only seven residues such as Tyr231, Phe235, Ile253, Ala302, Cys304, Glu363, and Arg364 forming a shallow cavity centered at (−0.01, 17.67, −6.31), indicative of a secondary or transient interaction site. Similarly, Pocket 3 was identified as a minor cavity with the lowest score (1.18), probability (0.011), and conservation (0.246), consisting of five residues (Leu213, Tyr231, Cys304, Ser306, and Glu363) centered at (−5.07, 20.39, −6.17). The small size and low conservation of Pockets 2 and 3 suggest limited suitability for high-affinity ligand binding. The comparative analysis clearly identifies Pocket 1 as the principal catalytic and ligand-binding pocket of CASP4, providing a robust structural basis for subsequent molecular docking, virtual screening, and inhibitor design (Figure 11).

**Figure 11:**
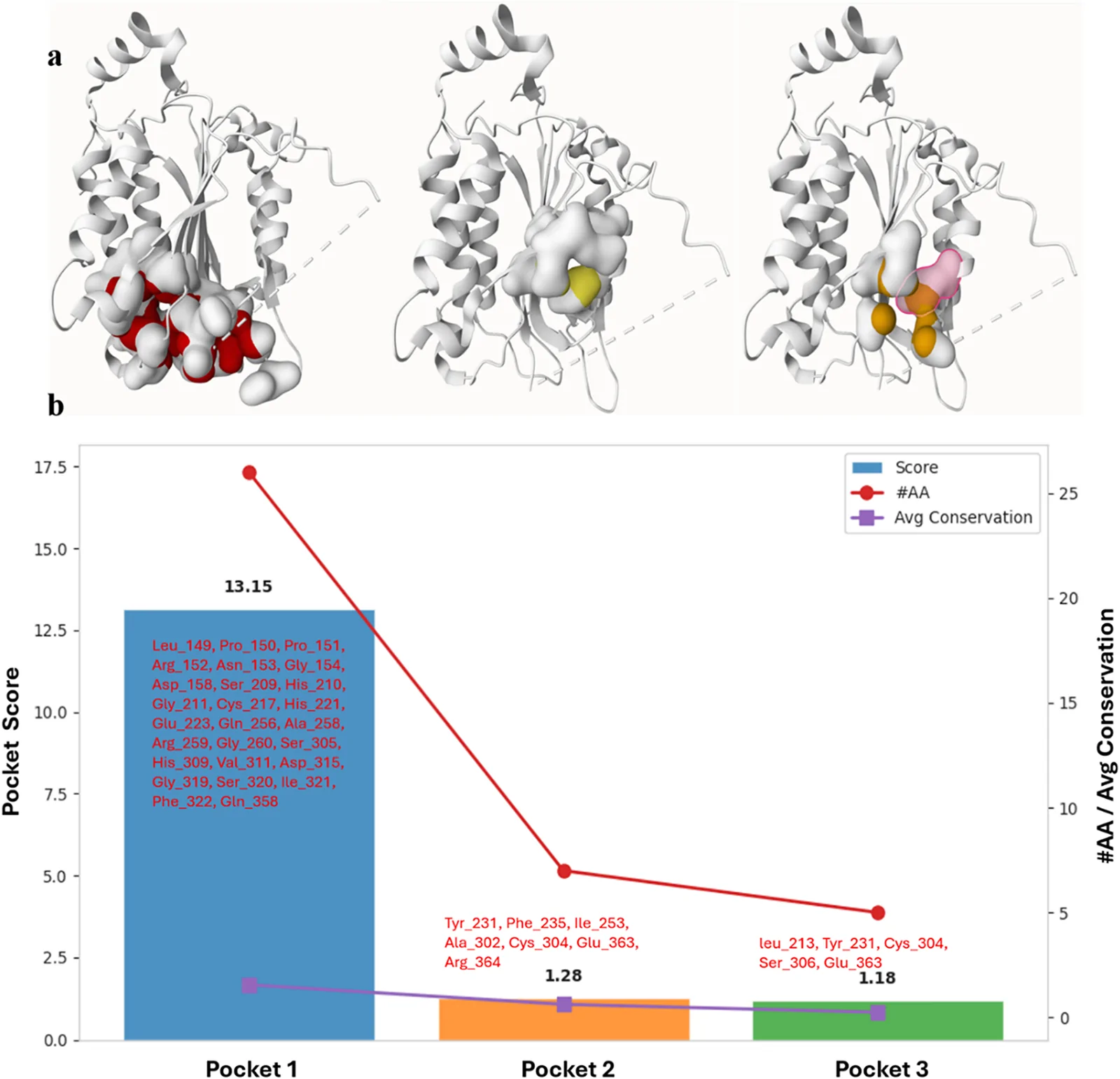
(a, b) Computational prediction of the CASP4 binding pocket and the key residues contributing to ligand binding.

### Molecular Docking Analysis

Molecular docking is most favorable computational approach for ligand-protein and protein–protein binding orientations.^20,89,90^ Docking energies ranged from −6.4 to −9.75 kcal/mol, indicating a broad spectrum of interaction strengths. Several compounds demonstrated notably stronger binding than the reference inhibitor Donepezil (−7.9 kcal/mol). The screened drugs DB01068 (−9.75 kcal/mol), DB09477 (−8.68 kcal/mol), DB05316 (−8.51 kcal/mol), DB08882 (−8.35 kcal/mol), and DB06202 (−8.0 kcal/mol) exhibited docking energies below the −8 kcal/mol threshold, suggesting favorable interaction profiles and enhanced binding stability within the CASP4 active site. These high-affinity candidates consistently outperformed lower-scoring molecules such as DB00519, DB01544, DB06203, and DB08897, which showed weaker binding energies in the −6.4 to −6.8 kcal/mol range. Overall, the docking results highlight a subset of top-performing compounds with strong predicted affinity toward CASP4, supporting their prioritization for subsequent MD simulations and free-energy evaluation (Figure 12).

**Figure 12:**
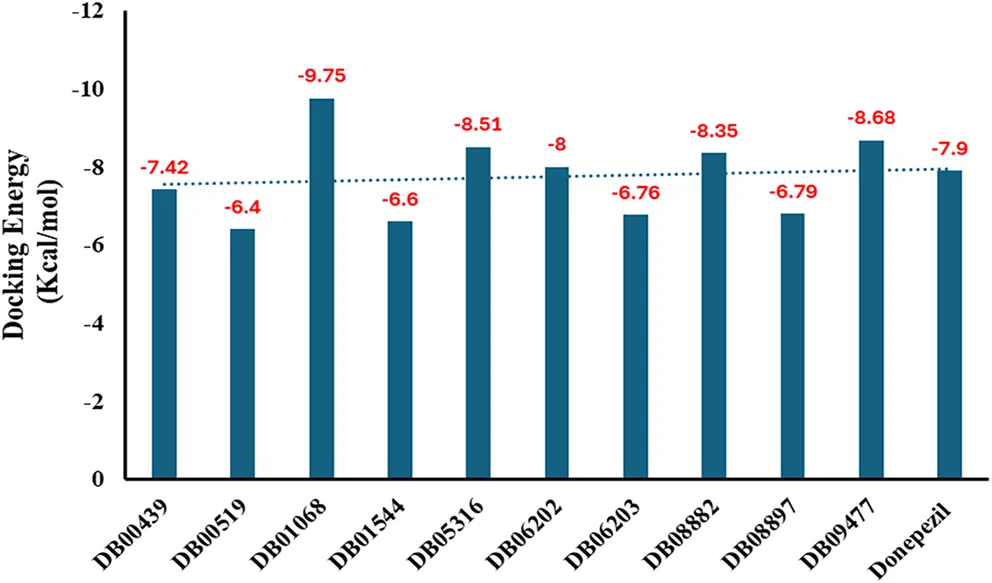
Docking energy profile of screened drugs against CASP4.

The superimposition of all docking complexes highlights a high degree of overlap in the binding orientation of the top-ranked ligands within the active site, supporting the reliability of the predicted poses for DB08882, DB05316, DB09477, and DB01544 as primary candidates. The longest-acting molecules DB00439, DB06203, and DB08897 occupy the same pharmacophoric region but show slightly greater divergence in peripheral substituents, consistent with subtle differences in their interaction networks and PK–PD profiles. The superimposed structures confirm that all drugs engage a common binding pocket and preserve key interaction features, while variations in side-chain extension and pocket filling likely account for the observed differences in predicted affinity and duration of action (Figure 13).

**Figure 13:**
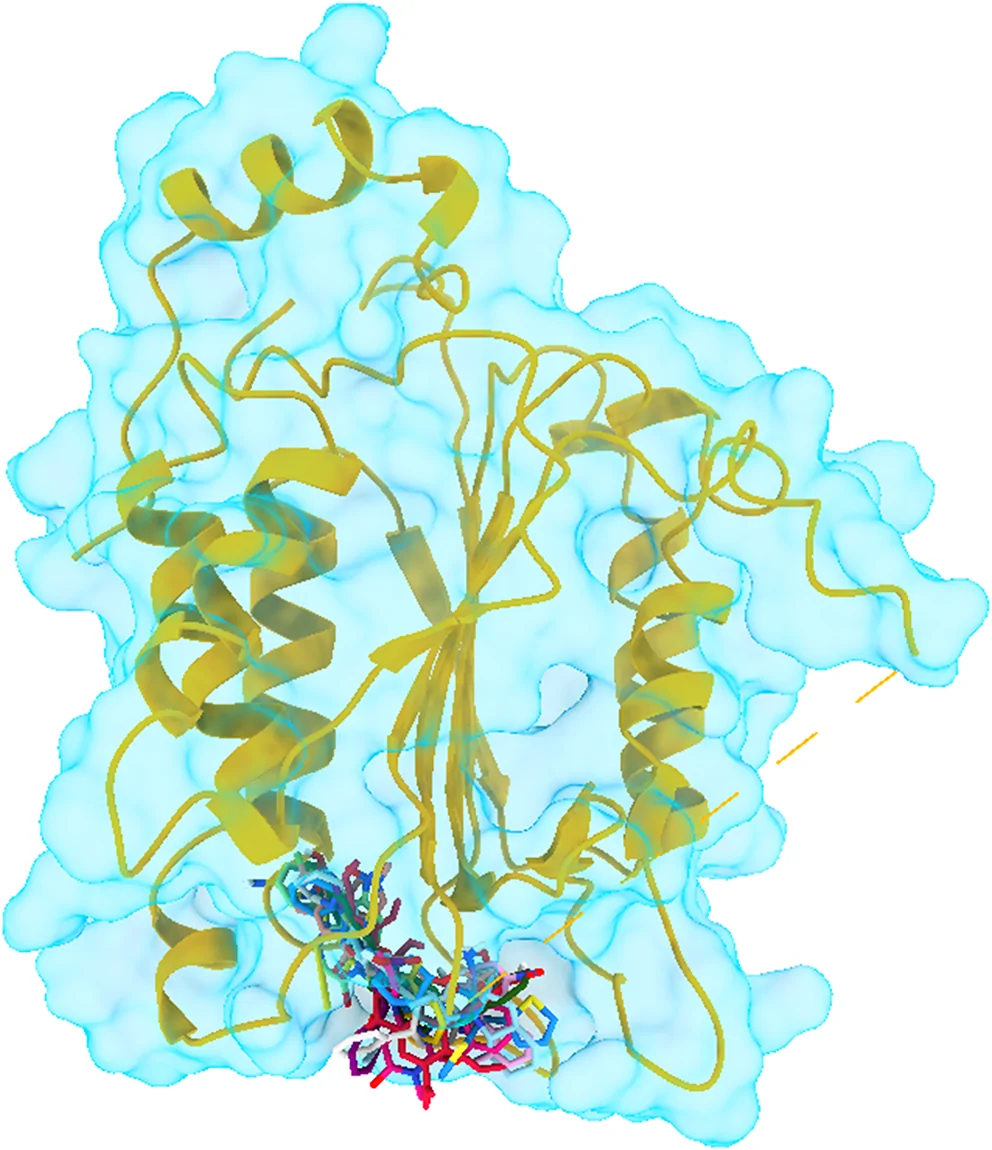
Superimposition of all screened docking complexes against CASP4.

### Binding Interaction Analysis

#### DB00439, DB00519, and DB01068 with CASP4 Interactions

The docking analysis of DB00439, DB00519, and DB01068 against CASP4 demonstrates stable and biologically meaningful binding within the catalytic pocket, supporting CASP4 as a promising therapeutic target in AD. Increasing evidence implicates CASP4 (and its murine homolog caspase-11) in neuroinflammation, pyroptosis, and non-canonical inflammasome activation, all of which contribute to AD pathology through microglial activation, amyloid-β induced inflammatory cascades, and neuronal injury.^91–93^ Recent transcriptomic and neuropathological studies further report elevated inflammatory caspase signaling in AD brains, reinforcing the therapeutic relevance of targeting inflammatory caspases such as CASP4.^94^

DB00439 exhibited stable anchoring within the CASP4 catalytic site through conventional hydrogen bonds with key residues including Ala258 and Arg152, along with polar interactions involving His210. Hydrogen bonding is essential for orienting ligands within cysteine-dependent protease active sites and contributes significantly to binding affinity and specificity.^95^ DB00439 engaged Ala258 and Arg152 through hydrogen bonds of 3.10 Å and 2.24 Å, respectively. Moreover, DB00439 also formed π–π stacking interactions with His210, His309 and π–alkyl contacts with Leu149 and Cys217, generating strong hydrophobic stabilization. The interaction pattern suggests that DB00439 achieves optimal complementarity within the CASP4 binding groove, potentially interfering with substrate access and catalytic activation.

DB00519 demonstrated a balanced interaction profile characterized by hydrogen bonding with Ala258, along with carbon hydrogen bonding to Gly260, reinforcing ligand orientation within the active site. DB00519 directly formed hydrogen bond with Ala258 with bond distance of 2.54 Å. Aromatic π–π interactions with His309 and hydrophobic contacts involving Leu149 and Val311 suggest strong occupancy of the S1/S2 subsites of CASP4. Hydrophobic packing and aromatic stacking are well recognized contributors to binding free energy and inhibitor potency in protease targets.^96,97^

DB01068 formed a strong hydrogen bond with His210 and His221 with bond length 2.81 and 2.99 Å, a residue proximal to the catalytic region, along with extensive π–alkyl and hydrophobic interactions involving Ile212, Cys217, and Leu149, respectively. The presence of π-sigma and aromatic stacking interactions further stabilized the ligand within the binding cleft. Such combined electrostatic and hydrophobic interactions are characteristic of high-affinity caspase inhibitors and are essential for blocking cysteine protease activity.^22^ The conserved engagement of hotspot residues (Ala258, Leu149, His210, Arg259) across all three complexes strengthens the reliability of the docking poses and suggests that these residues are critical for CASP4 inhibition. Neuroinflammation is a key driver of AD, with inflammasome-related caspases promoting amyloid-β and tau pathology.^92,94^ CASP4-dependent pyroptosis intensifies neuronal injury, making CASP4 inhibition a rational therapeutic target. Strong hydrogen bonding, aromatic stacking, and hydrophobic fit suggest DB00439, DB00519, and DB01068 can stably bind the CASP4 catalytic pocket and potentially reduce its inflammatory activity. These results justify follow-up through molecular dynamics, enzymatic assays, and AD-relevant neuroinflammation models (Figure 14).

**Figure 14:**
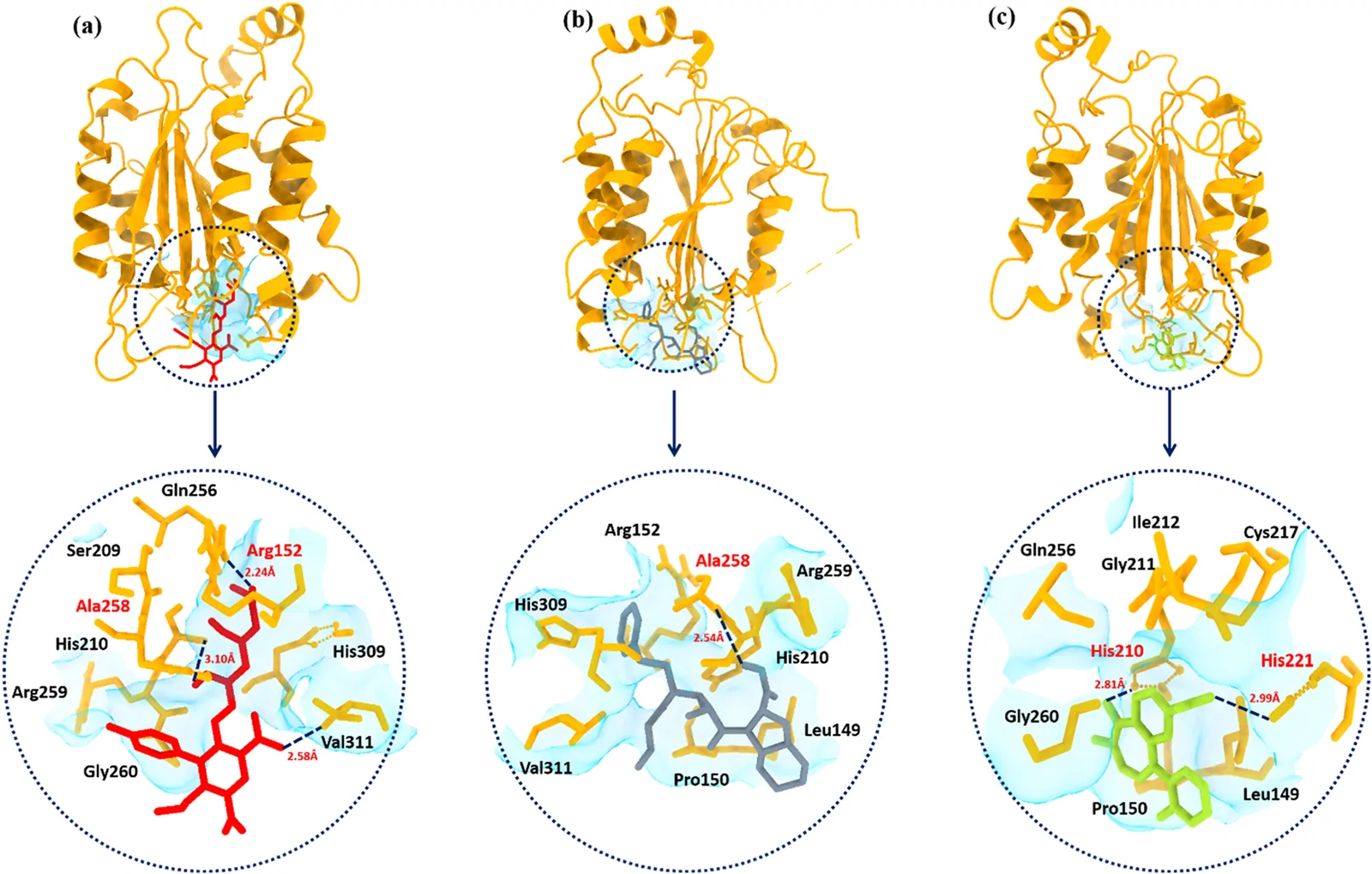
(a–c) The interactions profiles of DB00439 (a), DB00519 (b), and DB01068 (c) with CASP4 (light-orange ribbon). The screened drugs are represented in red, gray, and light green colors. The binding pocket is displayed as a sky-blue surface. Hydrogen-bond distances are indicated by dotted lines (Å), and interacting residues are labeled in red.

#### DB01544, DB05316, and DB06202 Interaction Analysis

In the DB01544-CASP4 complex, the ligand establishes two prominent conventional hydrogen bonds with Ala258 and Arg152 (2.74 and 2.30 Å, respectively), indicating strong polar anchoring within the active region. A halogen (fluorine) interaction is observed with Asn153, contributing to directional stabilization. Notably, a π–anion interaction with Asp315 is evident, suggesting electrostatic complementarity between the ligand’s aromatic system and the negatively charged residue. Additional π–π interaction with His309 and π–alkyl contacts with Val311 and Ala155 reinforce hydrophobic packing. The other surrounding residues such as Ala257, Ser209, Gln256, and Pro151 contribute van der Waals stabilization, collectively maintaining the ligand within the catalytic groove.

In the DB05316-CASP4 complex, the ligand displays a mixed electrostatic and aromatic interaction profile. A strong attractive charge interaction with Asp315 is present, indicating stabilization near the catalytic region. The ligand also forms halogen interactions with Asp158 and Gly319, enhancing binding directionality. A π–π interaction with His309 and π–alkyl interactions with Ala258 and His210 stabilize the aromatic scaffold within the pocket. The carbon hydrogen bonding with nearby residues and van der Waals contacts involving Ser209, Gly260, and Ala257 further support ligand accommodation within the active region of CASP4.

In the DB06202-CASP4 complex, the interaction pattern is predominantly hydrophobic and aromatic. Extensive π–alkyl interactions with Ala258, Leu149, and Val311 define the main stabilization forces. A carbon hydrogen bond with Gly260 is observed, while van der Waals contacts with Cys217, Gln256, and Gly211 support structural complementarity. The π-interactions with His210 and Pro150 further indicate aromatic stacking within the binding cleft. The comparative analysis showed that DB06202 ligand shows minimal direct electrostatic anchoring, relying instead on hydrophobic packing and π-mediated stabilization (Figure 15).

**Figure 15:**
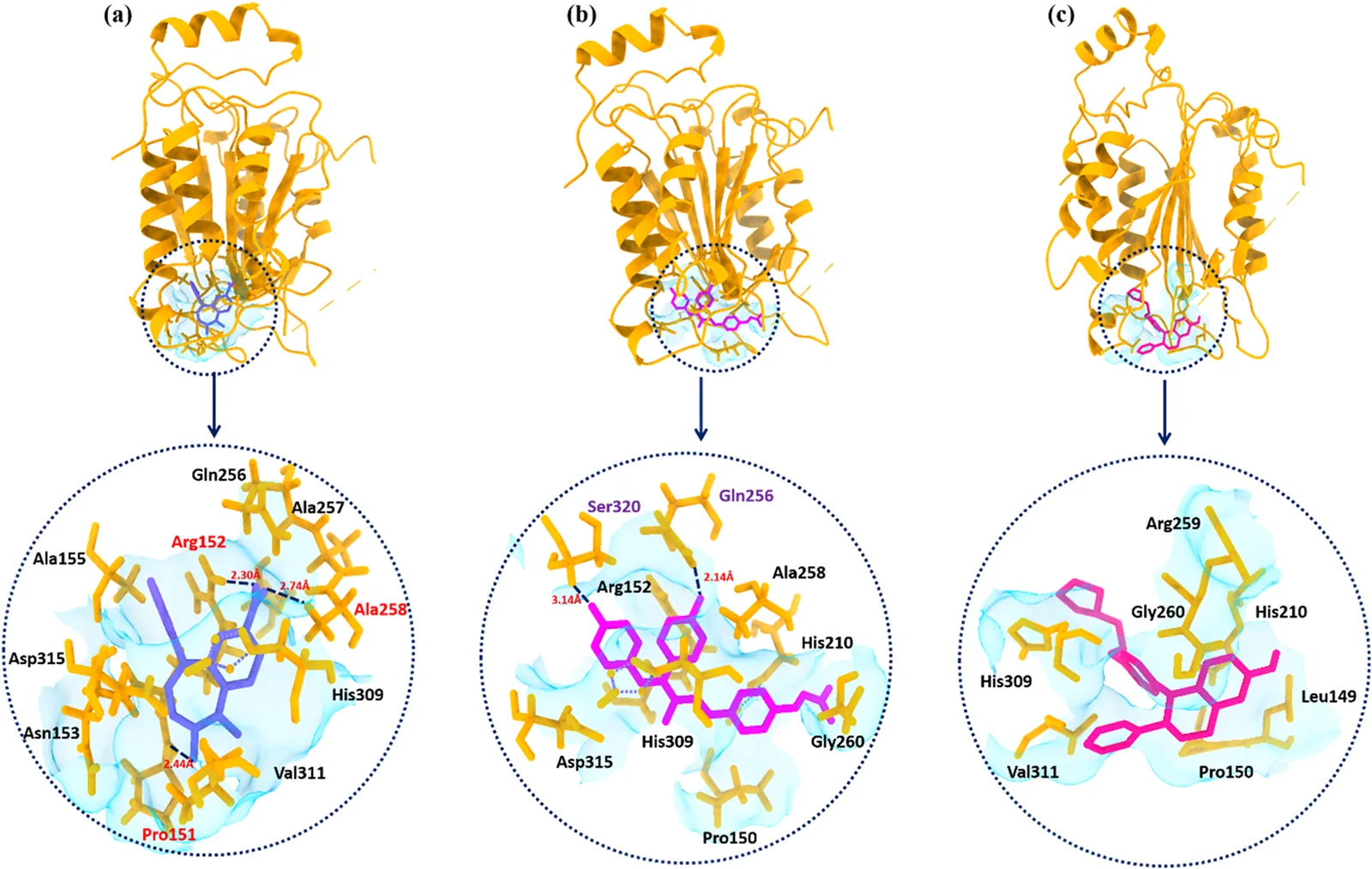
(a–c) Interactions of DB01544 (a), DB05316 (b), and DB06202 (c) with CASP4 (ribbon, light orange). The screened drugs are represented in blue, magenta, and pink colors. The binding pocket is depicted in sky blue surface representation. Hydrogen bond distances are shown as dotted lines (Å), with interacting residues labeled in red.

#### DB06203, DB08882, and DB08897 with CASP4 Interactions

DB06203 is stabilized primarily by hydrophobic packing against Val311, Pro309, Pro258, and Leu149, consistent with observed van der Waals and alkyl/π–alkyl interactions. The hydrogen bond has been observed at Ala258 (2.89 Å) which stabilizes one end of the ligand and orients the aromatic moiety toward the hydrophobic cleft. Additional weak contacts with Arg259, Arg152, Gly260, and His309 enlarge the interaction surface but do not form strong directional hydrogen bonds, suggesting that DB06203 relies predominantly on shape complementarity and dispersion forces for affinity. The lack of polar contacts to catalytically relevant residues may explain a comparatively lower binding strength and could translate into moderate inhibitory potency relative to the other two ligands.

In another docking complex, DB08882 exhibits a more extended aromatic framework that penetrates deeply into the active site and forms multiple π-stacking and π–alkyl interactions with surrounding residues (for example, His210, Pro150, Val311, and Ala258), generating a broad hydrophobic clamp around the ligand. Its poly-oxygenated core participates in several conventional and carbon–hydrogen bonds with Asp315, Asn153, Gly315, and Met321, which likely contribute substantial enthalpic stabilization and orient the ligand in a catalytically competent pose. The presence of charged and polar residues such as Arg152 and Ser329 provides additional electrostatic complementarity and helps to lock the scaffold through attractive charge and hydrogen-bond interactions. Overall, the dense network of directional hydrogen bonds combined with extensive aromatic and alkyl contacts suggests that DB08882 achieves the tightest binding and the most favorable interaction geometry among the tested compounds, making it a strong candidate for potent inhibition.

In DB08897-CASP4 docking complex, the drug adopts a bent conformation in the pocket, allowing its central aromatic system to engage in π–π and π–alkyl contacts with His210, His309, and Pro150, while its terminal rings extend toward more solvent-exposed regions. The ligand forms key conventional hydrogen bonds with Gly260 and His221, which anchor it near the same polar region occupied by DB08882, but with fewer hydrogen-bond donors/acceptors engaged. Additional van der Waals and alkyl interactions with Ser320, Leu149, and other neighboring residues help to stabilize the pose; however, the interaction map shows a less continuous hydrogen-bonding network compared with DB08882, implying somewhat reduced enthalpic contributions. Consequently, DB08897 is expected to display intermediate binding affinity: stronger and more specific than DB06203 because of its mixed aromatic polar contacts, yet less optimized than DB08882 due to the sparser polar interaction pattern (Figure 16).

**Figure 16:**
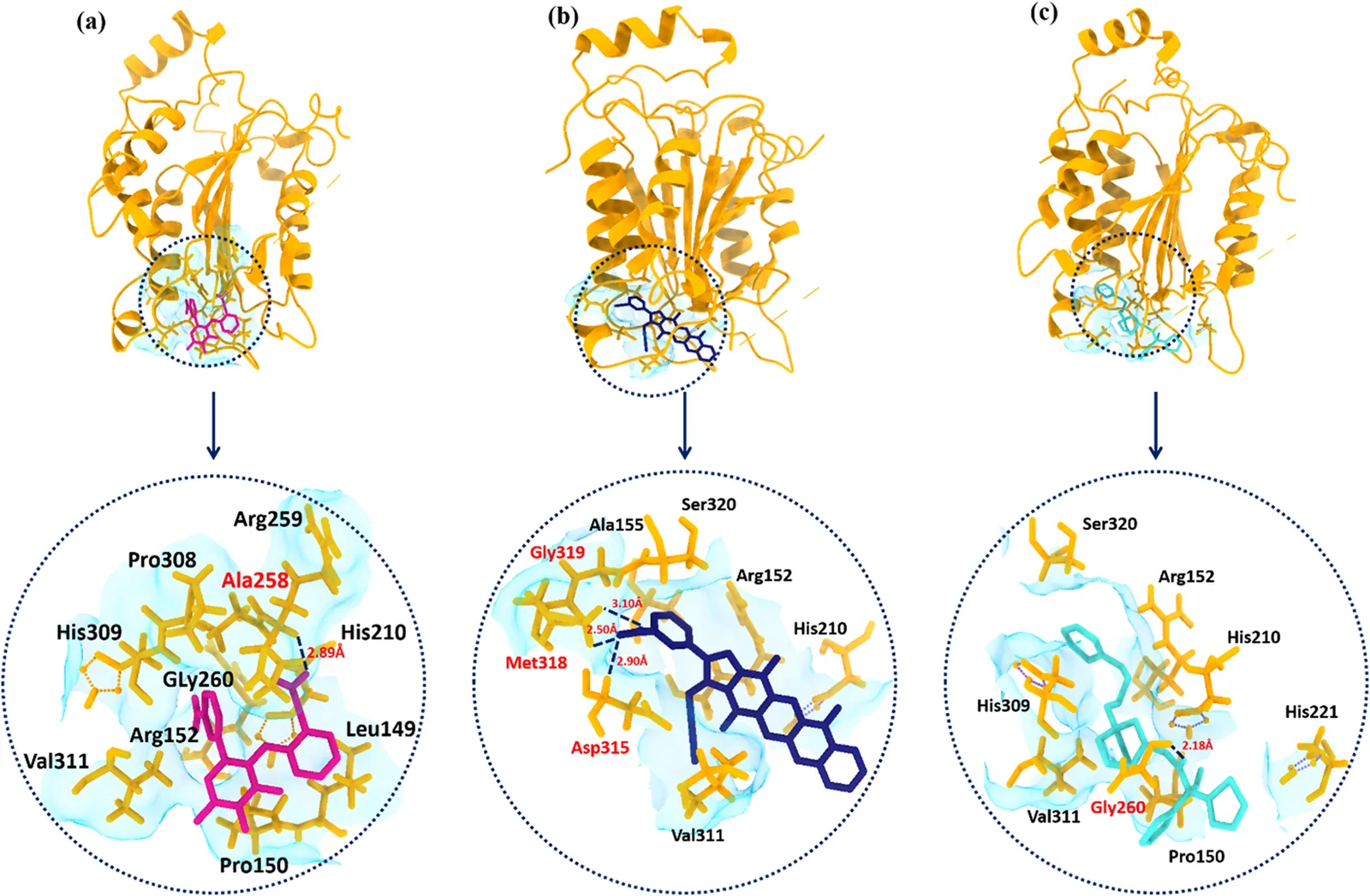
(a–c) DB06203 (a, light pink), DB08882 (b, dark blue) and DB08897 (c, cyan) bound to CASP4 (ribbon, light orange). Binding pocket shown as sky blue surface; hydrogen bonds indicated by dotted lines (Å), interacting residues labeled in red.

#### DB09477 and Donepezil Docking against CASP4

DB09477 fits snugly into the active pocket and is mainly stabilized by hydrophobic and van der Waals contacts with Pro309, Pro159, Val311, Ala155, Ala258, and Arg259. It forms conventional hydrogen bonds with Gly260 and Asp158, while additional carbon–hydrogen bonds to Asn153 and Gln256 further anchor the scaffold. Alkyl and π–alkyl interactions with Arg152 and neighboring non-polar residues broaden the contact surface but provide limited directionality, so the complex is interpreted as having moderate yet meaningful affinity. Donepezil adopts an extended conformation that allows its aromatic rings to be tightly clamped by π–alkyl and alkyl contacts with Val311, Pro150, His210, and Arg259. Key conventional and carbon–hydrogen bonds with Asn153, Ala258, and His309 fix the orientation of the heterocyclic core, while a π–cation interaction with Arg152 and a π–sulfur contact with Cys217 add extra electrostatic stabilization. Together, these interactions give donepezil a denser and more optimized binding network than DB09477, consistent with its role as a high-affinity reference ligand and explaining its expected superiority in docking scores (Figure 17). The 2D depiction has been depicted in Figure 18.

**Figure 17:**
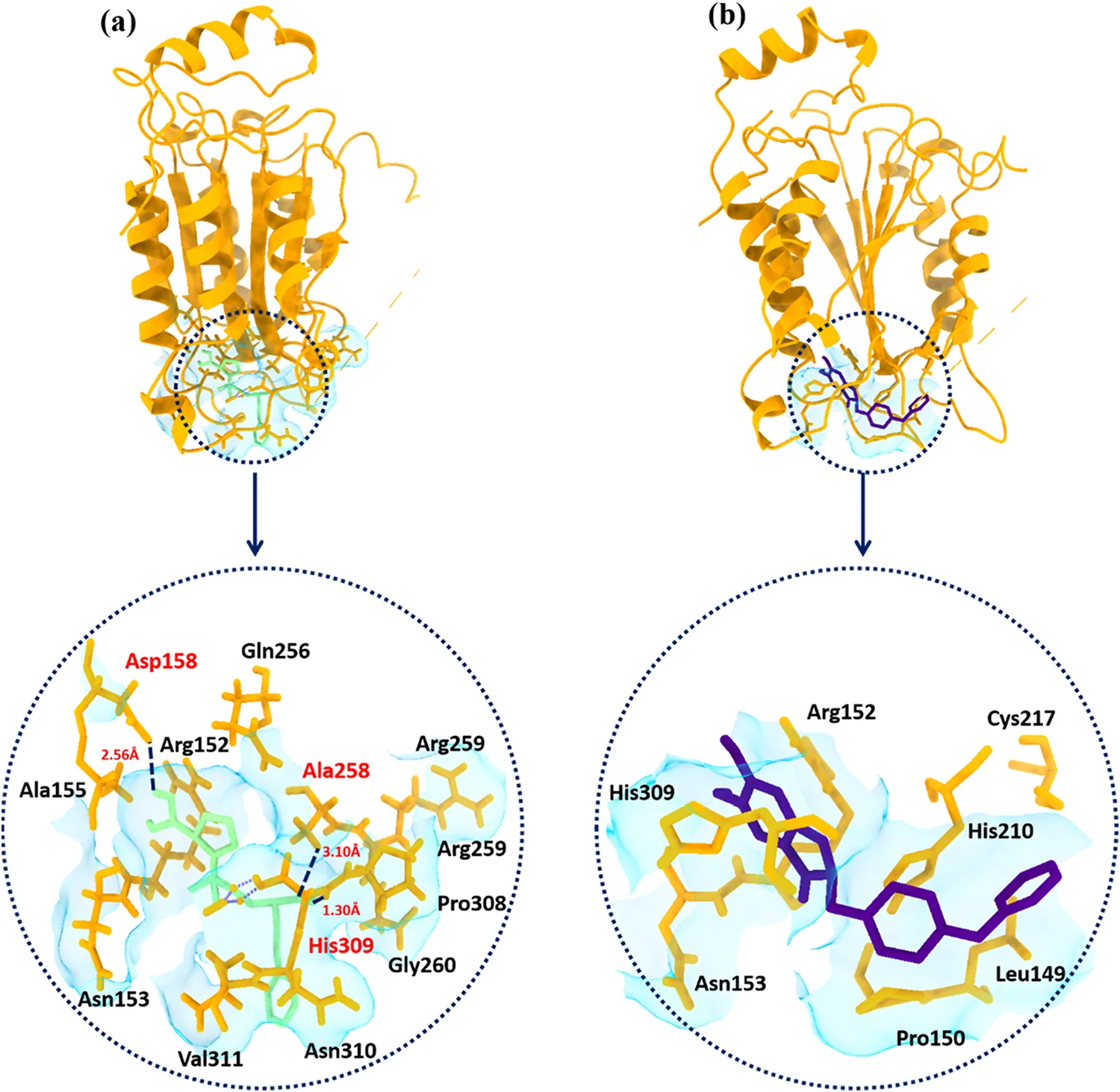
(a, b) DB09477 (light green), and donepezil (dark blue) in complex with CASP4 (light orange ribbon, sky blue pocket surface), respectively. Dotted lines indicate hydrogen bond distances (Å); residues labeled in red.

**Figure 18:**
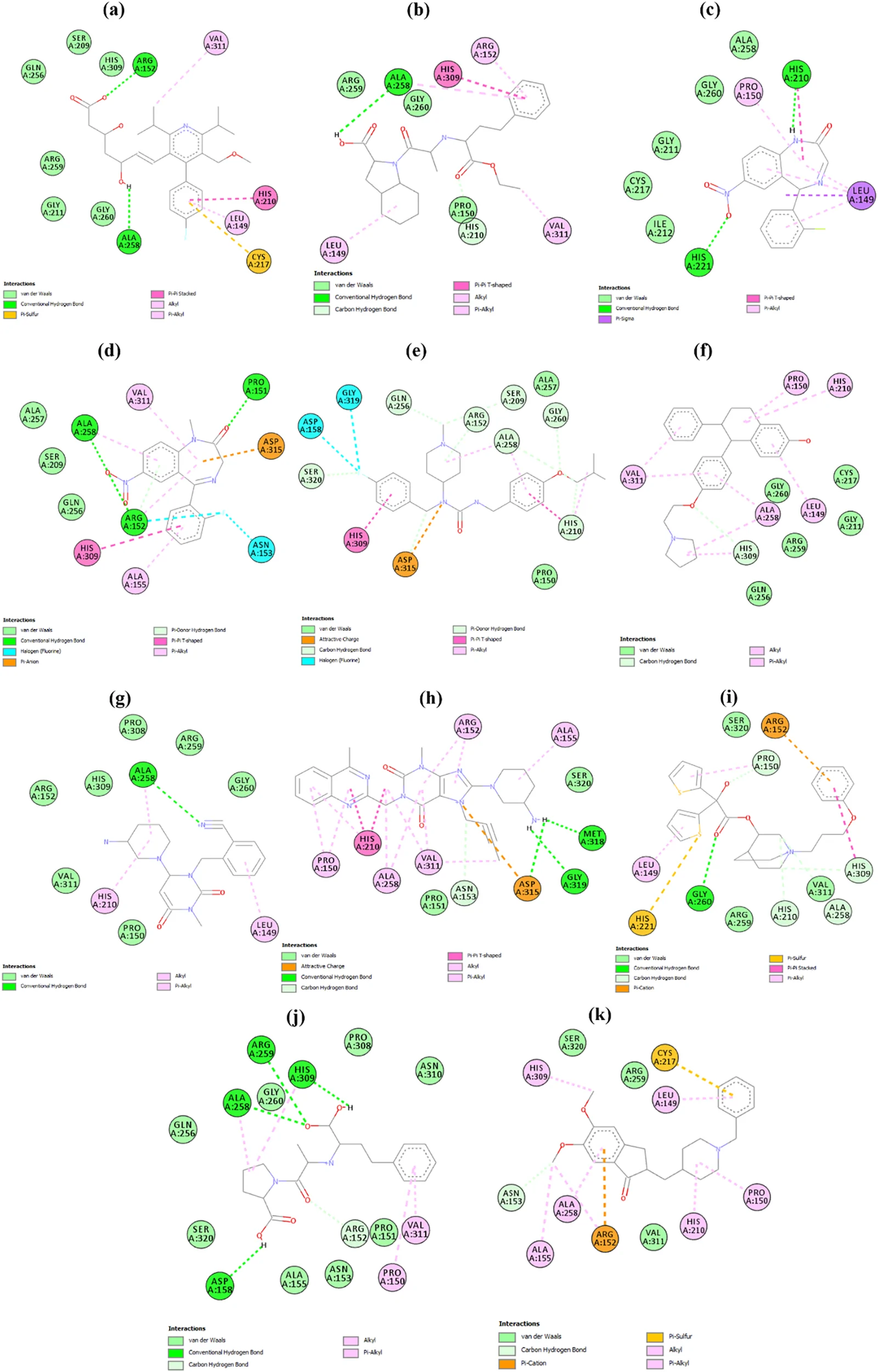
(a–k) Depiction of docking complexes of DB00439, DB00519, DB01068, DB01544, DB05316, DB06202, DB06203, DB08882, DB08897, DB09477, and donepezil, respectively, showing their interactions with CASP4 alongside the standard drug.

### MD Simulations Analysis

#### Root Mean Square Deviation (RMSD) Analysis

The structural stability of five drug-target complexes (DB00519, DB01068, DB05316, DB06202, and DB08882) was evaluated using backbone RMSD over 100-nanosecond molecular dynamics (MD) simulations performed in triplicates (R1, R2, and R3) and compared directly against Donepezil as a reference standard. Across all test platforms, Donepezil maintained a highly consistent, baseline trajectory with a stable average RMSD of 0.23 nm. Among the candidate molecules, complex DB00519 (A) demonstrated the most favorable and tightly converged structural profile, with all three replicates equilibrating smoothly after 20 ns to maintain a low overall average RMSD of 0.22 nm. This exceptional convergence indicates that DB00519 successfully anchors the protein into a rigid, highly stable conformation, mimicking or slightly outperforming the reference drug. Such minimal structural fluctuation typically correlates with optimized complementary interactions within the active pocket and a lower likelihood of premature ligand dissociation. In contrast, the remaining candidates displayed varying degrees of conformational flexibility and divergence, highlighting distinct structural adaptations upon ligand binding. Complex DB01068 (B) revealed a split dynamic profile; while its R1 and R3 replicates remained exceptionally rigid at 0.21 nm, the R2 replicate underwent a major structural transition after 40 ns, driving its individual average to 0.27 nm and peaking above 0.35 nm. This pronounced behavior suggests that DB01068 forces the protein to cross an energy barrier to sample an alternative conformational state, which may signify an induced-fit binding mechanism or localized structural strain. Moderate flexibility was observed in complex DB05316 (C), where stable R2 and R3 runs were offset by an upward drift in the R1 trajectory that fluctuated between 0.25 and 0.30 nm. Complex DB06202 (D) showed the greatest overall geometric instability, driven heavily by its R3 replicate which spiked markedly to ∼0.33 nm near the 60 ns mark before settling at a high average of 0.26 nm. Finally, complex DB08882 (E) yielded a unique, decentralized profile where the R1 trajectory stayed highly rigid near 0.18 nm, while the R3 counterpart experienced an early upward expansion peaking near 0.29 nm before stabilizing. The stark variations observed between individual replicates in panels B, D, and E underscore a critical methodological takeaway: the absolute necessity of multi-replicate MD runs in computer-aided drug design. The classical molecular dynamics is inherently stochastic, relying on a single trajectory could easily lead to an inaccurate assessment of a complex’s true thermodynamic stability. Therefore, capturing these diverse, alternative trajectories through triplicate runs ensures that local energy minimum is accounted for, providing a far more rigorous, biologically accurate, and reproducible foundation for selecting the most promising lead compounds. Although the RMSD traces exhibit noticeable fluctuations, these variations correspond to expected conformational adjustments of CASP4 during ligand binding rather than loss of structural integrity. Across the three independent 100 ns replicates, each complex reaches a stable RMSD plateau after the initial relaxation phase. The average RMSD values (0.22–0.30 nm) fall within the typical range reported for ligand-bound caspases. Complementary analyses including RMSF, *R*_g_, and hydrogen-bond occupancy further confirm that the protein backbone remains compact and that ligand interactions are preserved throughout the simulations. Thus, the RMSD behavior reflects normal dynamic flexibility rather than instability (Figure 19).

**Figure 19:**
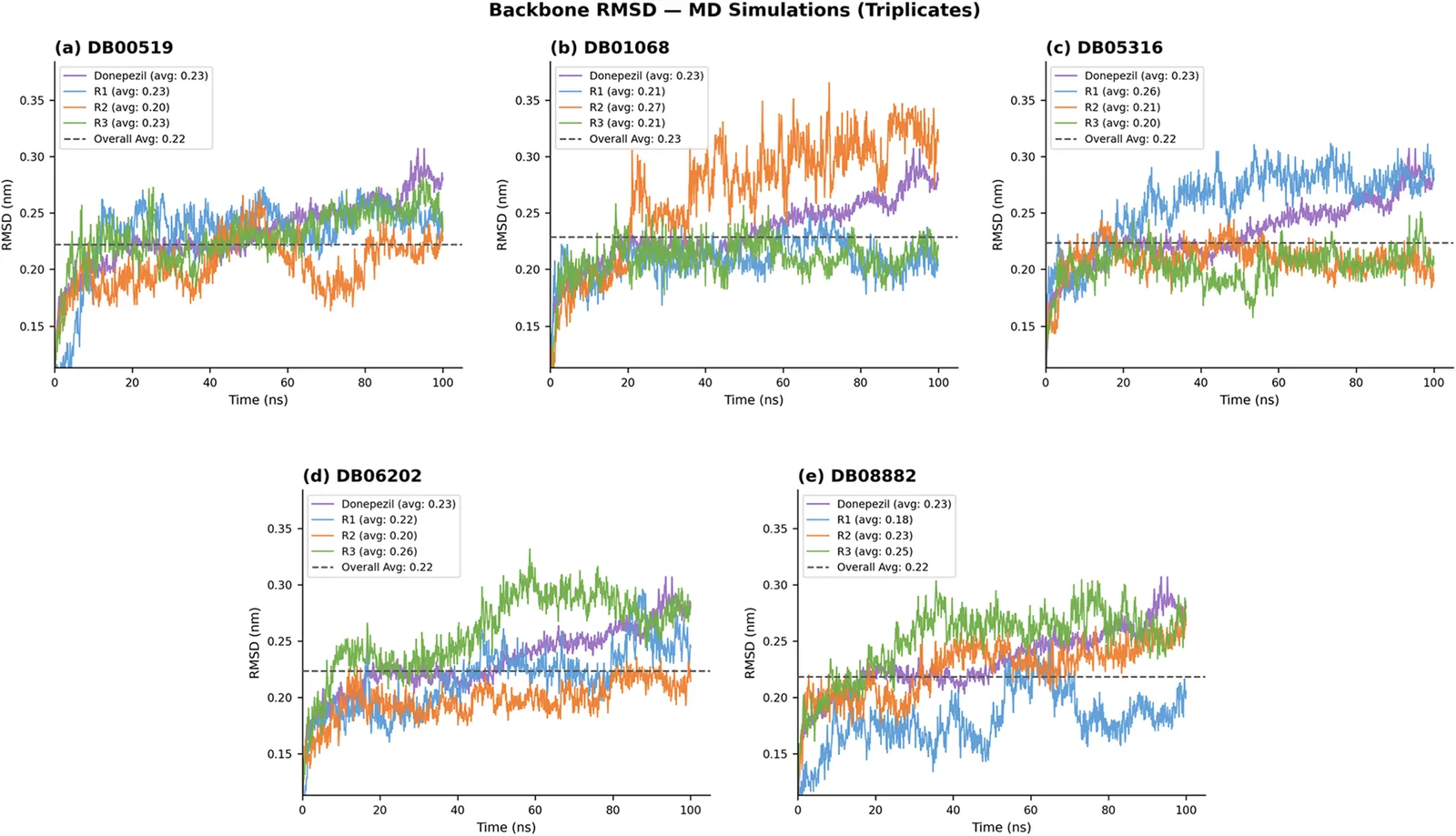
(a–e) Backbone root mean square deviation (RMSD) profiles of protein-ligand complexes over 100 ns molecular dynamics simulations. The structural stability of five target complexes (a) DB00519, (b) DB01068, (c) DB05316, (d) DB06202, and (e) DB08882 is evaluated across independent triplicate trajectories designated as R1 (blue solid line), R2 (orange solid line), and R3 (green solid line). Each panel displays the comparative control trajectory of the reference drug Donepezil (orange flat trace; average: 0.23 nm) alongside the overall combined average RMSD of the respective test system (horizontal black dashed line).

#### Root Mean Square Fluctuation

The local residual mobility and structural flexibility of the target protein upon binding five candidate molecules (DB00519, DB01068, DB05316, DB06202, and DB08882) were characterized via Root Mean Square Fluctuation (RMSF) analysis across triplicate trajectories (R1, R2, and R3). The reference drug Donepezil established a baseline of controlled stability with an average fluctuation of 0.105 nm. The RMSF profiles for all complexes shared a conserved pattern of rigid structural cores (troughs keeping below 0.10 nm) interrupted by highly localized flexible domains (peaks exceeding 0.30 nm), primarily corresponding to loop regions on the protein surface. Among the candidates, complex DB00519 (a) demonstrated an average RMSF ranging from 0.097 to 0.115 nm, exhibiting a highly unique, elevated plateau in the R1 replicate between residues 250 and 290. This sustained localized flexibility suggests a loop rearrangement specific to that trajectory, though the remaining peaks tightly mirrored Donepezil. Complex DB01068 (b) yielded highly rigid average values (0.092 to 0.110 nm) but experienced a massive fluctuation spike reaching above 0.50 nm near residue 310 in the R2 replicate. This extreme localized mobility perfectly correlates with the significant backbone transition observed for DB01068 R2 in the preceding RMSD analysis, confirming that the macro-conformational shift was driven by an intense localized loop opening or domain movement. For complex DB05316 (c), fluctuations were well-contained (averaging 0.081 to 0.112 nm), though its R1 replicate demonstrated sharp, elevated peaks near residues 225, 315, and 360, pointing to localized thermal instabilities. Complex DB06202 (d) showed elevated baseline noise (up to 0.118 nm average in R1), characterized by sharp, erratic spikes across the entire residue sequence specifically around residues 225, 290, and 315 reflecting an overall increase in structural looseness. Lastly, complex DB08882 (e) maintained remarkably uniform trajectories across its triplicates (averaging 0.101 to 0.110 nm), save for a shared, prominent peak near residue 225, where all three runs spiked identically to ∼0.45 nm, suggesting an intrinsic hinge movement in that specific loop region regardless of the replicate run. When mapped together, the consistent rigidity observed across most complexes within the troughs indicates that the core catalytic or binding framework of the protein remains structurally preserved. The sharp, variable peaks among the replicates (particularly in panels b and d) highlight the dynamic nature of outer surface loops as they adapt to accommodate different ligand structures. Crucially, the localized divergence in replicate runs such as the isolated residue 310 spike in DB01068 (R2) validates the multi-replicate simulation strategy, demonstrating that specific loop-opening events are stochastic and would be entirely missed in standard single-trajectory studies (Figure 20).

**Figure 20:**
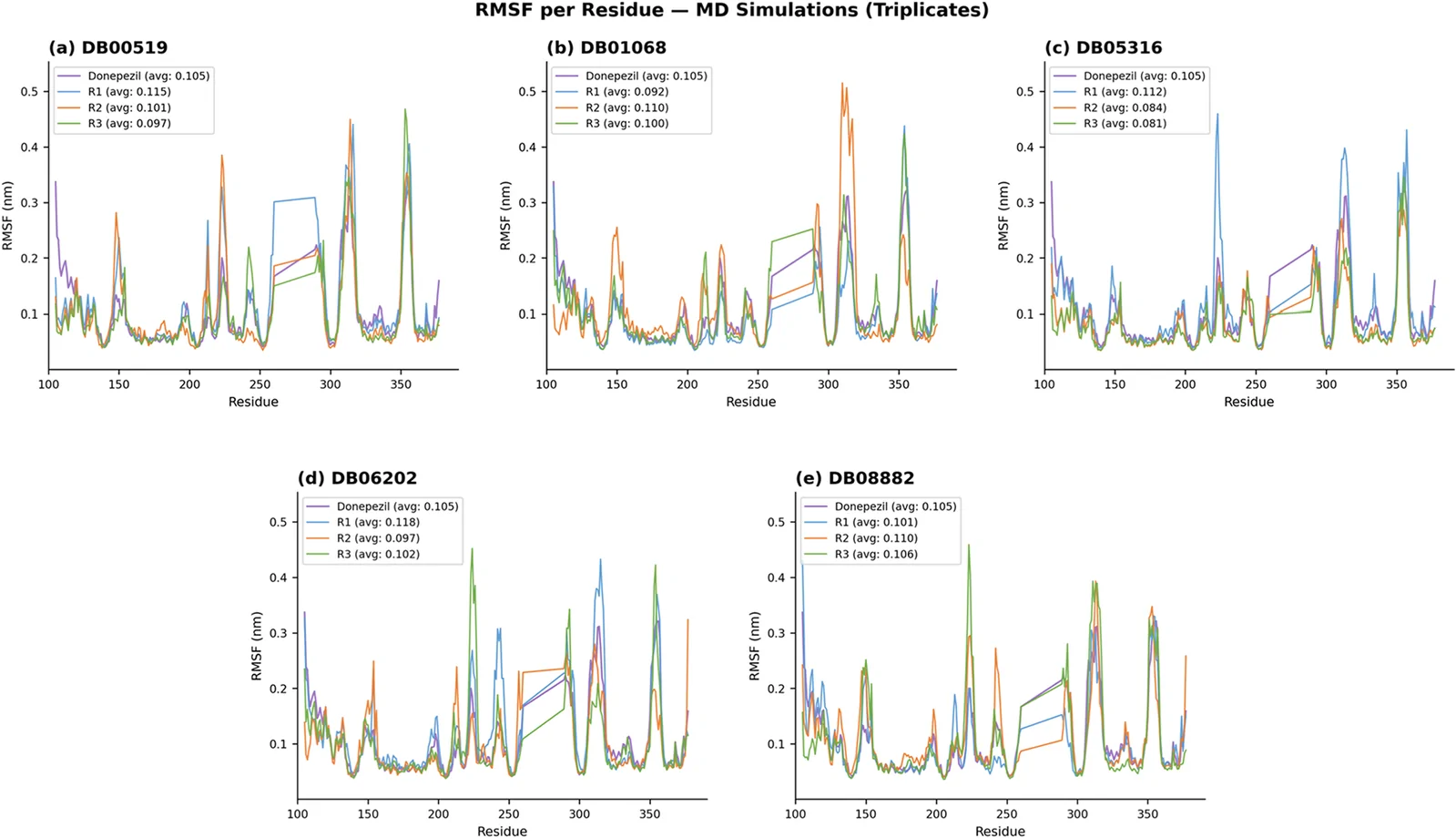
(a–e) Root mean square fluctuation (RMSF) profiles per residue for protein-ligand complexes. Localized residual flexibility of five test complexes (a) DB00519, (b) DB01068, (c) DB05316, (d) DB06202, and (e) DB08882 is plotted across residues 100–380 for triplicate trajectories: R1 (blue), R2 (orange), and R3 (green). The purple trace serves as comparative reference control (Donepezil; average RMSF: 0.105 nm). Individual replicate averages are provided in the respective legends.

#### Solvent-Accessible Surface Area (SASA) Analysis

The structural compactness, folding behavior, and surface exposure of the target protein upon binding five candidate molecules (DB00519, DB01068, DB05316, DB06202, and DB08882) were investigated through Solvent Accessible Surface Area (SASA) analysis across 100 ns triplicate trajectories. The reference drug Donepezil established a highly stable, baseline surface exposure profile with a constant average SASA value of 132.46 nm^2^. In comparison, the test candidate complexes showed distinct alterations in surface area, signifying variable levels of global structural expansions or compactions during the simulations. Complex DB00519 (a) maintained an elevated overall average SASA of 134.27 nm^2^. This expansion was driven notably by its R3 replicate (green, avg: 136.18 nm^2^), which remained elevated throughout the timeline, suggesting that the binding of this ligand induces a slightly more open or extended protein conformation. Conversely, complex DB01068 (b) demonstrated a balanced and steady profile with a combined average of 132.40 nm^2^, closely matching the Donepezil control. While the R2 replicate (orange) experienced a sharp drop to ∼125 nm^2^ early on, it equilibrated back to the baseline after 50 ns, confirming that the structural transition observed in previous RMSD/RMSF steps resolved into a native-like, compact equilibrium. The most profound compaction was observed in complex DB05316 (c), which yielded the lowest overall average SASA at 129.80 nm^2^. This decline was starkly defined by the R2 replicate (orange, avg: 125.75 nm^2^) and the R3 replicate (green, avg: 130.00 nm^2^), both of which trended significantly below the control baseline over the entire 100 ns duration. This steady compression indicates a dense, highly compact packing of the protein core, which may restrict internal cavity movements. In contrast, complex DB06202 (d) showed high trajectory overlapping and a stable overall average of 132.62 nm^2^, reflecting uniform, well-conserved protein dimensions across all three independent runs. Lastly, complex DB08882 (e) exhibited a clear expansion trend with the highest overall average SASA of 134.93 nm^2^, fueled by its R2 and R3 runs fluctuating upward to peak near 144 nm^2^, revealing a highly dynamic and solvent-exposed protein outer shield. The combined SASA findings reveal that while DB01068 and DB06202 preserve standard protein density, DB05316 induces unique structural contraction, and DB00519 and DB08882 shift the protein toward a more expanded layout. These shifts are biologically vital, as variations in solvent exposure alter the water-shell dynamics around the active site, directly impacting ligand binding kinetics and thermodynamic stability. The noticeable trajectory splits particularly the intense structural compression unique to the R2 run of DB05316 further highlight the value of triplicate simulations in successfully capturing rare, macro-conformational collapsing or unfolding pathways (Figure 21).

**Figure 21:**
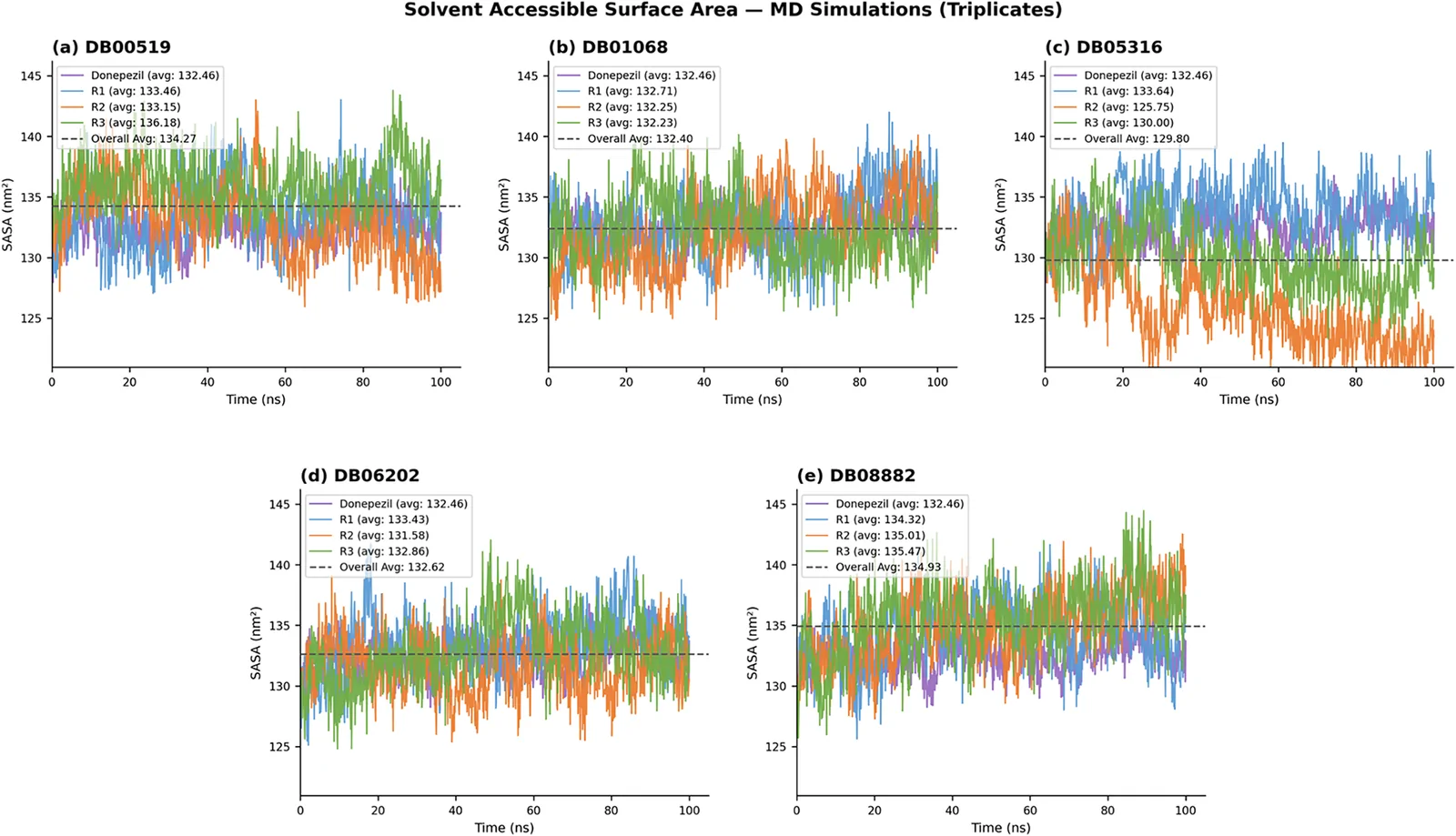
(a–e) Solvent accessible surface area (SASA) profiles protein-ligand complexes over 100 ns MD timelines. Total surface exposure variations for (a) DB00519, (b) DB01068, (c) DB05316, (d) DB06202, and (e) DB08882 are evaluated using independent triplicate trajectories: R1 (blue), R2 (orange), and R3 (green). The steady purple trace represents the reference standard control (Donepezil; average SASA: 132.46 nm^2^), while the black dashed line indicates the group’s global average.

#### Protein Compactness Analysis Using *R*_g_ Analysis

The global hydrodynamic volume, structural compactness, and packing density of the target protein upon binding five candidate molecules (DB00519, DB01068, DB05316, DB06202, and DB08882) were analyzed via the Radius of Gyration (*R*_g_) across 100 ns triplicate simulations. The reference control Donepezil maintained a remarkably rigid and steady profile, maintaining a constant average (*R*_g_) value of 1.83 nm. Fluctuations or shifts in (*R*_g_) directly indicate whether a ligand maintains the protein’s native compact fold or induces structural expanding/collapsing pathways. Complex DB00519 (a), DB01068 (b), and DB06202 (d) all exhibited an overall average (*R*_g_) of 1.83 nm, perfectly matching the Donepezil reference standard and indicating highly conserved macro-structural stability. Within these systems, subtle variations were captured: the R2 replicate (orange) of DB00519 showed a minor compaction trend down to ∼1.81 nm after 60 ns, while the R2 replicate of DB01068 showed an upward fluctuation peaking near 1.86 nm toward the end of the simulation. This temporary expansion in DB01068 matches the previous open-loop dynamics seen in its corresponding RMSD and RMSF profiles.

The most notable deviations occurred in complexes C and E, which altered the protein’s global dimensions. Complex DB05316 (c) yielded a compressed overall average (*R*_g_) of 1.82 nm. This global compaction was heavily driven by its R2 replicate (orange), which dropped sharply and stably hovered between 1.80 and 1.81 nm across the entire timeline. This structural compaction strongly correlates with the lower solvent exposure observed in the preceding SASA analysis, confirming that DB05316 stabilizes a highly compressed, tightly packed protein core. Conversely, complex DB08882 (e) showed an expansion trend with the highest overall average (*R*_g_) of 1.84 nm. Its R2 (orange) and R3 (green) replicates experienced a steady upward drift after 40 ns, peaking near 1.87 nm. This expansion highlights a loosening of the protein matrix, allowing greater flexibility and higher solvent accessibility. The (*R*_g_) analysis confirms that the tertiary structure of the target enzyme is dynamically responsive to different ligand chemotypes. The stable, overlapping trajectories of DB00519, DB01068, and DB06202 imply that these compounds preserve the native, functional architecture of the protein receptor. The unique compression induced by DB05316 suggests a highly stable, tightly bound state that could potentially lock the binding pocket, whereas the expansion seen in DB08882 indicates a highly flexible conformation. Furthermore, the distinct, separated trajectory lines, especially the sustained compaction of the R2 run in panel C, reaffirm the necessity of running triplicate MD simulations to avoid false structural conclusions from single-run stochastic anomalies (Figure 22).

**Figure 22:**
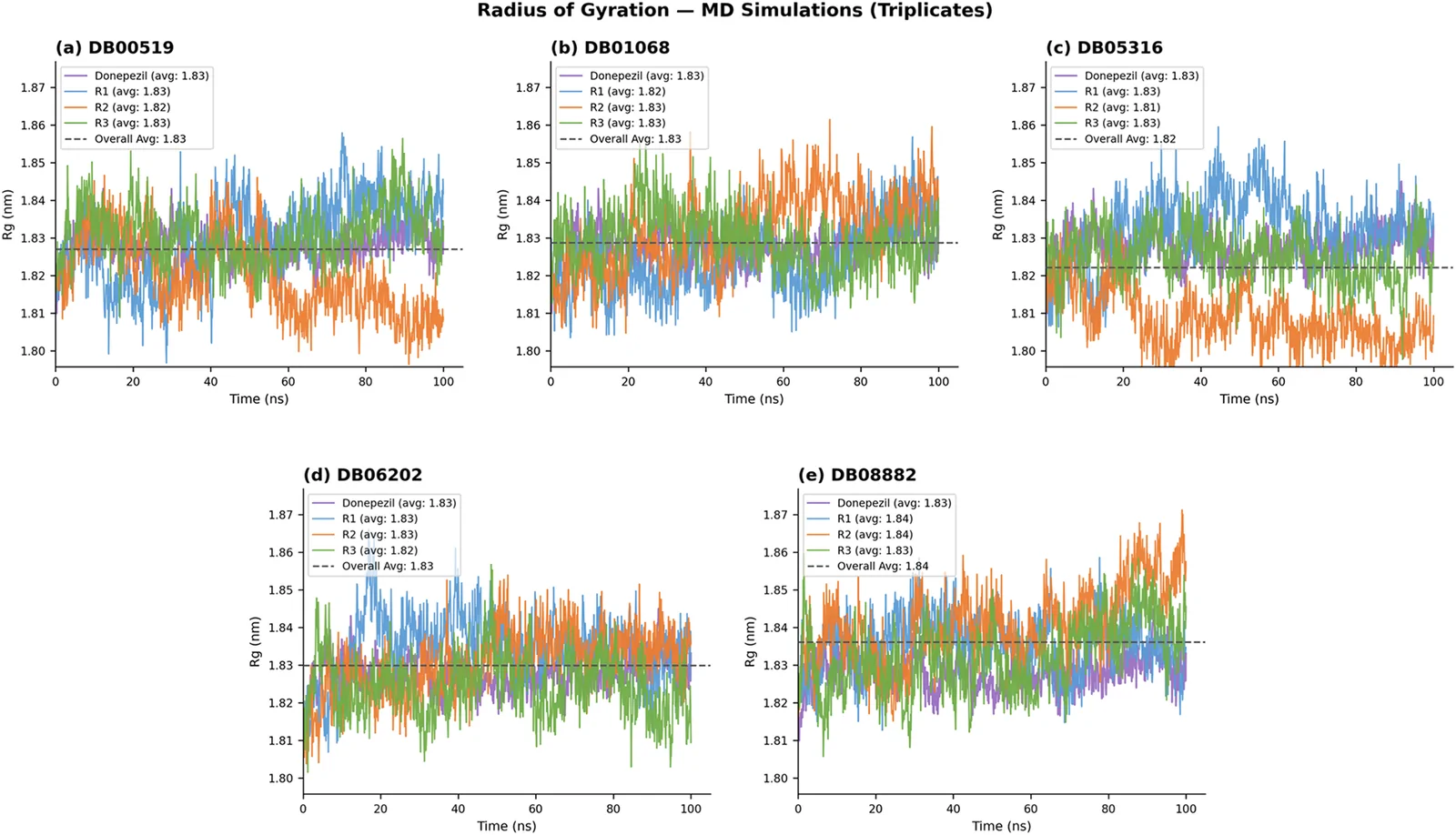
(a–e) *R*_g_ profiles of protein-ligand complexes over 100 ns MD simulations. Global structural compactness variations for (a) DB00519, (b) DB01068, (c) DB05316, (d) DB06202, and (e) DB08882 are evaluated using independent triplicate trajectories: R1 (blue), R2 (orange), and R3 (green). The steady purple trace represents the reference control (Donepezil; average (*R*_g_): 1.83 nm), and the horizontal black dashed line indicates the global average for each system. Individual simulation averages are provided in the respective legends.

#### Protein-Ligand Distance Analysis

The binding durability, pocket retention, and potential unbinding events of five candidate molecules (DB00519, DB01068, DB05316, DB06202, and DB08882) were analyzed by tracking the Protein-Ligand Center of Mass (COM) distance across 100 ns triplicate simulations. The reference drug Donepezil maintained an exceptionally rigid, steady trajectory, hovering at an average distance of 1.94 nm. Persistent, low-amplitude COM distances signify tight, continuous anchored binding within the receptor pocket, while sudden expansions or continuous drifts reveal ligand displacement or pocket instability. The complex DB00519 (a) demonstrated strong anchoring capabilities with an overall average COM distance of 1.93 nm, slightly lower than Donepezil. Its R2 (orange, avg: 1.88 nm) and R3 (green, avg: 1.71 nm) replicates stayed completely flat, indicating a tightly bound state. However, the R1 replicate (blue) drifted to ∼2.5 nm between 40 and 80 ns before returning toward the pocket, capturing a temporary ligand reorientation event. Conversely, complex DB01068 (b) experienced a clear binding failure, yielding the highest overall average distance of 2.38 nm. This was driven by the R3 replicate (green, avg: 2.96 nm), which completely dislodged from its original position early on, hovering at 3.0 nm before undergoing a massive spike to 5.0 nm at 55–60 ns. This represents a complete ligand unbinding or unassisted pocket exit. The complex DB05316 (c) yielded the lowest overall average distance at 1.87 nm, reflecting superb binding affinity. This stable configuration was anchored by replicates R1 (blue, avg: 1.74 nm) and R3 (green, avg: 1.69 nm), which remained exceptionally flat and well beneath the control baseline. This persistent closeness perfectly aligns with the global compression and reduced solvent accessibility observed in the preceding SASA and *R*_g_ metrics. For complex DB06202 (d), significant spatial divergence was noted (overall avg: 2.13 nm). Replicate R2 (orange) and R3 (green) continuously drifted upward to hover near 2.5 nm after 40 ns, indicating that the ligand shifted out of the optimal catalytic center. Lastly, complex DB08882 (e) showcased robust structural equilibration (overall avg: 1.98 nm). Aside from a brief, transient fluctuation to 3.0 nm at the 35 ns mark in the R3 run (green), all three trajectories tightly converged back to the 1.94 nm reference line for the remainder of the timeline. The COM distance profiles reveal crucial insights into the real-time binding mechanics of these therapeutic agents. The flawless retention seen in DB05316 and DB00519 suggests that these compounds form strong, persistent intermolecular bonds (such as hydrogen bonding and hydrophobic packing) that successfully resist thermal fluctuations. In contrast, the unbinding event in DB01068 (R3) and the structural drifts in DB06202 reveal a lack of geometric complementarity or weak electrostatic anchoring within the binding domain. These structural exits and shifts emphasize the power of multi-replicate testing: relying on a single trajectory could have falsely classified DB01068 as a stable binder based on its R1 run, highlighting how triplicate simulations protect against false-positive drug discovery selections (Figure 23).

**Figure 23:**
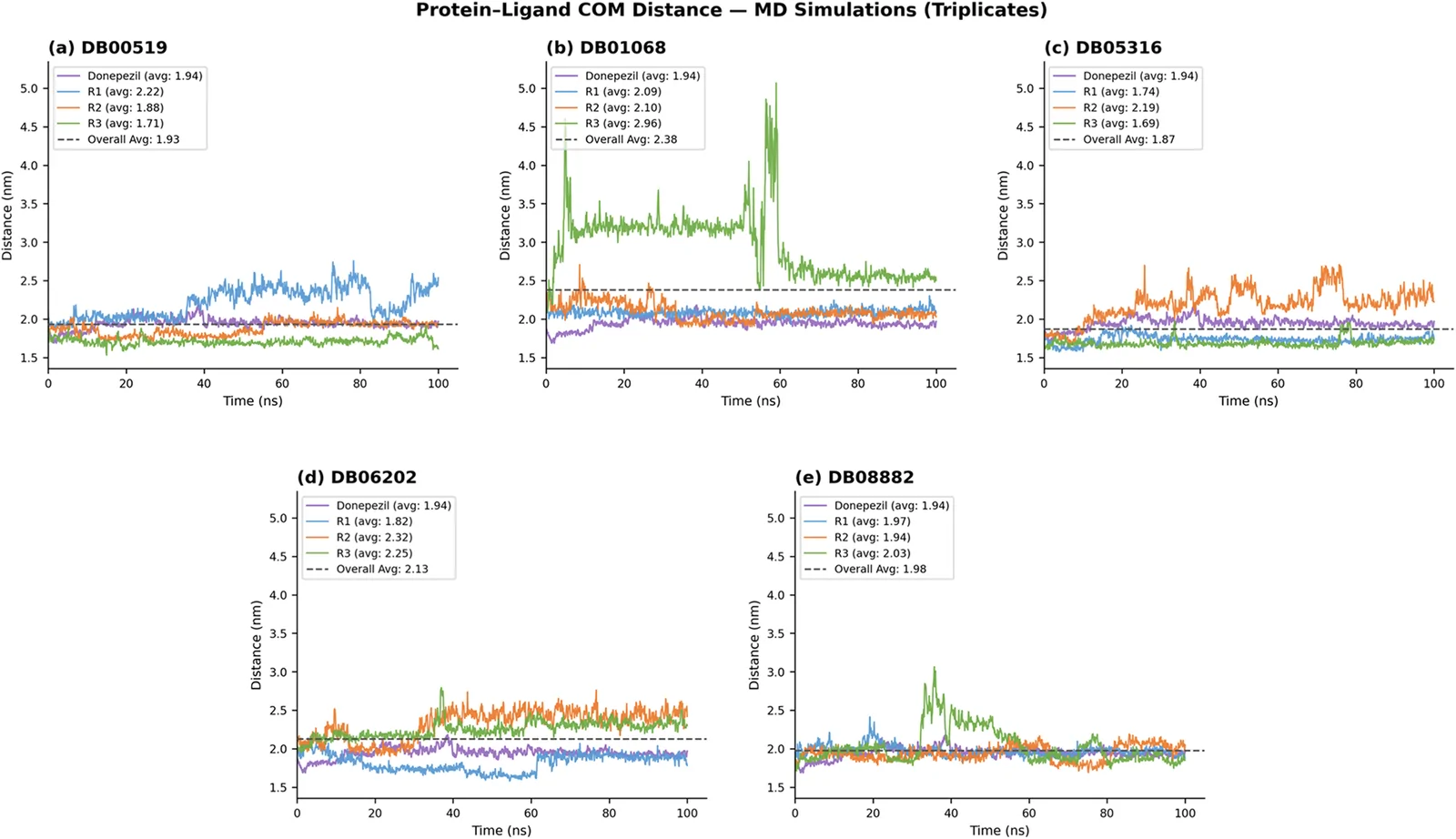
(a–e) Protein-ligand center of mass (COM) distance profiles over 100 ns MD simulations. Positional binding stabilities for (a) DB00519, (b) DB01068, (c) DB05316, (d) DB06202, and (e) DB08882 are evaluated inside the receptor pocket using triplicate trajectories: R1 (blue), R2 (orange), and R3 (green). The steady purple trace represents the reference control (Donepezil; average distance: 1.94 nm), and the horizontal black dashed line represents the global system average.

Furthermore, hydrogen bonds can exhibit cooperative effects, where one interaction subtly influences the strength of others. Prior work, shows that the energetic contribution of multiple hydrogen bonds is not strictly additive and that the counting argument may hold only in specific structural environments.^98^ In our study, the selected potential captures the dominant pairwise interactions, which is appropriate given the localized nature of the binding features examined. However, incorporating cooperative scaling behavior in future work may provide a more complete description of multi-bond coupling in complex systems.

#### Total MD Simulation Energy Analysis

The global thermodynamic equilibrium, energy conservation, and system stability of five drug-target complexes (DB00519, DB01068, DB05316, DB06202, and DB08882) were analyzed by tracking total potential and kinetic energy profiles across 100 ns triplicate simulations. The reference control Donepezil demonstrated a perfectly stable, flat trajectory averaging −615,903.55 kJ/mol. Across all panels, the characteristic, steep downward plunge during the initial 0 to 2 ns phase followed by a vertical spike after 98 ns reflects the transition between energy minimization/equilibration phases and data production boundaries, ensuring the systems were successfully relaxed. The complex DB00519 (a) maintained an overall average total energy of −615,882.40 kJ/mol, showing highly converged triplicate paths that tightly match Donepezil, indicating an energy-optimized system. Conversely, complex DB01068 (b) stabilized at a significantly higher overall average energy profile of −609,296.49 kJ/mol, driven by elevated values across all three replicates (R1, R2, R3 staying around −609,000 kJ/mol). This notable upward shift in the energy floor indicates that the DB01068 system rests at a higher, less favorable thermodynamic state, which explains the structural transitions and ligand unbinding events captured in the preceding COM distance and RMSD assessments. The complex DB05316 (c) also showed elevated total system energy (overall avg: −611,298.58 kJ/mol). Though its energy states were higher than the control, the individual trajectories remained well-behaved and flat throughout the production run. The most energetically stable complexes were (d) and (e). Complex DB06202 (d) stabilized below the control baseline with an overall average energy of −616,360.75 kJ/mol, indicating a highly relaxed system architecture. The lowest energy state was achieved by complex DB08882 (e), reaching a profound thermodynamic minimum with an overall average of −617,276.49 kJ/mol, driven uniformly by all three tightly overlapping replicate traces. The total energy profiles validate that all five MD production setups adhered strictly to the laws of conservation of energy under NPT ensembles, showing no anomalous drift or energy leaks. The distinct variations in the steady-state baselines reflect the internal thermodynamic impact of the different ligand structures on the protein-solvent network. The deeper, more negative global energy floors of DB08882 and DB06202 reveal a highly optimized, favorable structural state. Meanwhile, the higher energy plateaus of DB01068 confirm a less optimal atomic packing, providing a clear thermodynamic explanation for its weak pocket retention and tendency to undergo structural shifts (Figure 24a–e).

**Figure 24:**
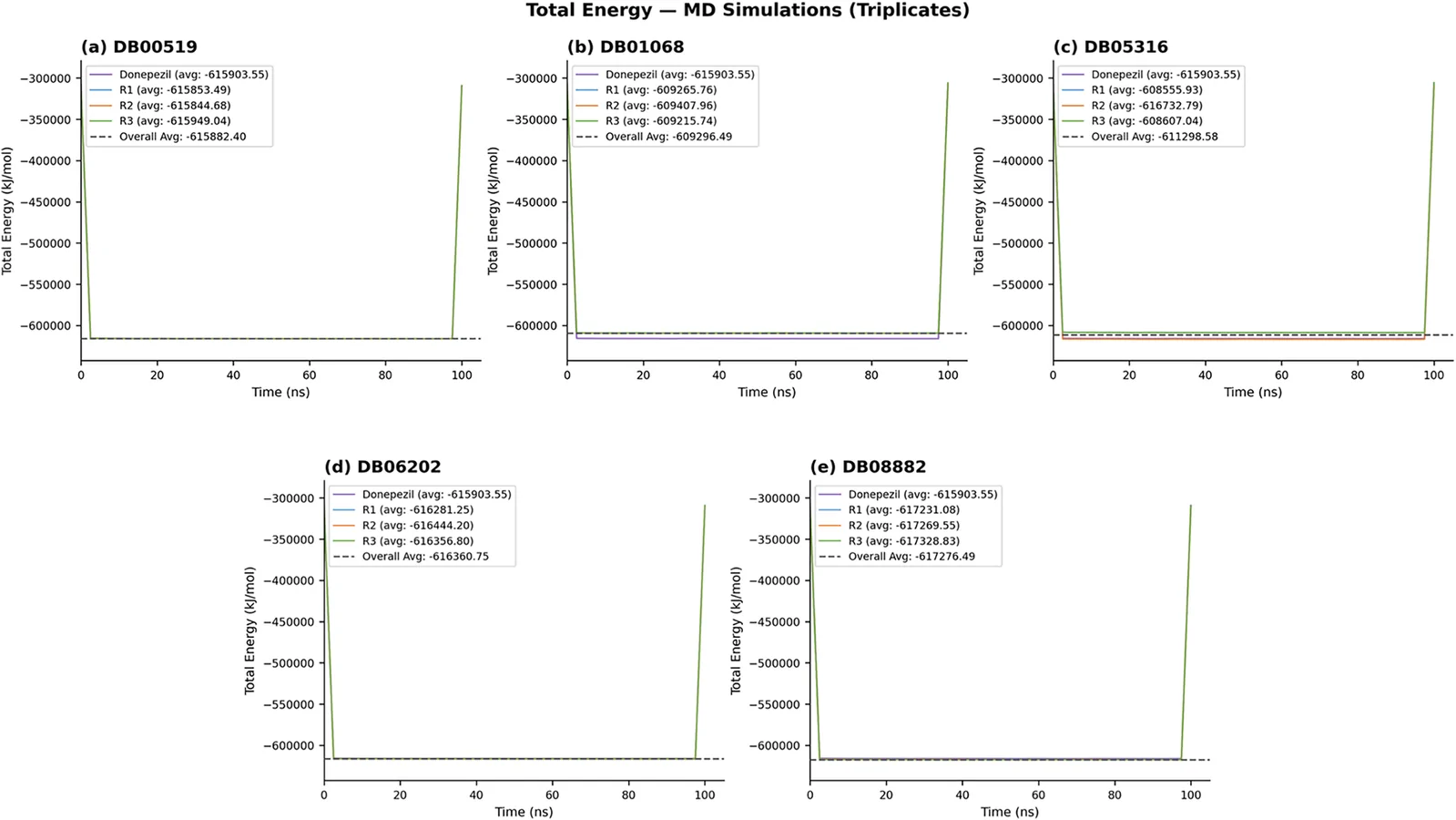
(a–e) Total system energy profiles of protein-ligand complexes over 100 ns MD simulations. Thermodynamic stability and energy conservation for (a) DB00519, (b) DB01068, (c) DB05316, (d) DB06202, and (e) DB08882 are recorded in kilojoules per mole (kJ/mol) using independent triplicate trajectories: R1 (blue), R2 (orange), and R3 (green). The steady purple trace represents the reference control (Donepezil; average energy: −615,903.55 kJ/mol), and the horizontal black dashed line highlights the global group average.

#### Dynamic Simulation Energy Evaluation

The combined energy table provides a comprehensive overview of the thermodynamic behavior of all ligands–protein complexes across the three independent MD simulation replicates (R1, R2, and R3). Reporting replicate-resolved values is essential for ensuring statistical robustness and reproducibility in MD-based binding stability assessments, as recommended in recent computational chemistry literature.^99^ For each compound Donepezil, DB00519, DB01068, DB05316, DB06202, and DB08882 the table summarizes potential energy, kinetic energy, total energy, temperature, pressure, and density, enabling direct comparison of the stability and equilibration quality across replicates. Across all systems, the temperature remained tightly centered around 303 K, consistent with stable thermostat performance under NPT conditions. Density values also remained within the expected range for solvated biomolecular systems (1033–1034 kg/m^3^), indicating proper barostat behavior and system equilibration.

The total energy values provide the most direct indicator of ligand-protein complex stability. Donepezil, used as the standard reference, exhibited highly consistent total energy values across replicates (−615,914 to −615,917 kJ/mol), reflecting stable binding and excellent reproducibility. Among the screened compounds, DB08882 demonstrated the most favorable energetic profile, with consistently more negative total energy values across all replicates (−617,215 to −617,307 kJ/mol). This suggests a stronger and more stable interaction with the protein target, consistent with the principle that lower total energy correlates with enhanced structural stability and binding affinity in MD simulations.^100^ DB06202 and DB00519 also displayed moderately favorable energy values with relatively low replicate variability, indicating stable binding behavior. In contrast, DB01068 and DB05316 exhibited higher total energies and greater variability, particularly DB05316, which showed substantial replicate-to-replicate fluctuations. Such variability may reflect conformational heterogeneity, insufficient stabilization of the ligand within the binding pocket, or sampling of multiple metastable states phenomena commonly observed in less stable ligand protein complexes.^101^ Overall, the combined table highlights DB08882 as the most energetically stable candidate, followed by DB06202 and DB00519, while DB01068 and DB05316 appear less stable based on their higher and more variable total energy values. These findings align with established MD-based evaluation strategies, where replicate-averaged energetic metrics provide a reliable indicator of ligand stability and binding quality (Table 5).

**Table 5:** MD Simulation Generated Energy Values in Triplicates

Drugs	Replicas	Potential (kJ/mol)	Kinetic (kJ/mol)	Total Energy (kJ/mol)	Temperature (K)	Pressure (bar)	Density (kg/m^3^)
Donepezil	R1	–758470	142556	–615914	303.117	0.067	1033.38
	R2	–758448	142561	–615887	303.128	0.429	1033.36
	R3	–758479	142563	–615917	303.132	0.761	1033.71
DB00519	R1	–758542	142696	–615846	303.139	0.968	1033.42
	R2	–758536	142692	–615844	303.131	0.267	1033.40
	R3	–758627	142694	–615933	303.135	0.650	1033.43
DB01068	R1	–751949	142694	–609254	303.128	1.017	1033.54
	R2	–752095	142696	–609399	303.132	1.121	1033.84
	R3	–751953	142701	–609252	303.143	0.716	1033.47
DB05316	R1	–751280	142715	–608565	303.132	0.790	1033.42
	R2	–759432	142712	–616720	303.125	0.892	1033.51
	R3	–751327	142713	–608614	303.126	0.619	1033.55
DB06202	R1	–758942	142653	–616289	303.128	0.680	1033.30
	R2	–759086	142652	–616434	303.126	0.382	1033.55
	R3	–759001	142650	–616351	303.122	0.438	1033.33
DB08882	R1	–759937	142722	–617215	303.131	0.013	1033.57
	R2	–759988	142726	–617262	303.138	0.787	1033.54
	R3	–760031	142724	–617307	303.135	1.081	1033.56

#### MMPBSA Analysis

Figure 25 illustrates the MM/PBSA binding free energy (Δ*G*_bind_) ranking of six compounds evaluated using the Poisson–Boltzmann (PB) implicit solvation model, revealing thermodynamically favorable binding interactions (Δ*G*_bind_ <0) across all simulated complexes. Compound DB05316 demonstrated the highest predicted binding affinity with a Δ*G*_bind_ of −21.37 kcal/mol, closely followed by DB00519 at −20.92 kcal/mol. Crucially, the top three database candidate compounds DB05316, DB00519, and DB06202 (−19.13 kcal/mol)—all outscored the reference drug Donepezil (−17.06 kcal/mol). This enhanced performance suggests that these novel candidates may engage in more optimal electrostatic or hydrophobic interactions within the receptor’s binding pocket compared to the standard Donepezil. Conversely, DB08882 (−13.53 kcal/mol) and DB01068 (−13.32 kcal/mol) exhibited the weakest binding profiles, indicating less complementary structural fit or suboptimal desolvation penalties. While the standard deviation error bars indicate localized conformational flexibility during the molecular dynamic’s trajectories particularly for DB06202 and Donepezil the clear energetic separation firmly establishes DB05316 and DB00519 as the most promising lead scaffolds for future optimization.

**Figure 25:**
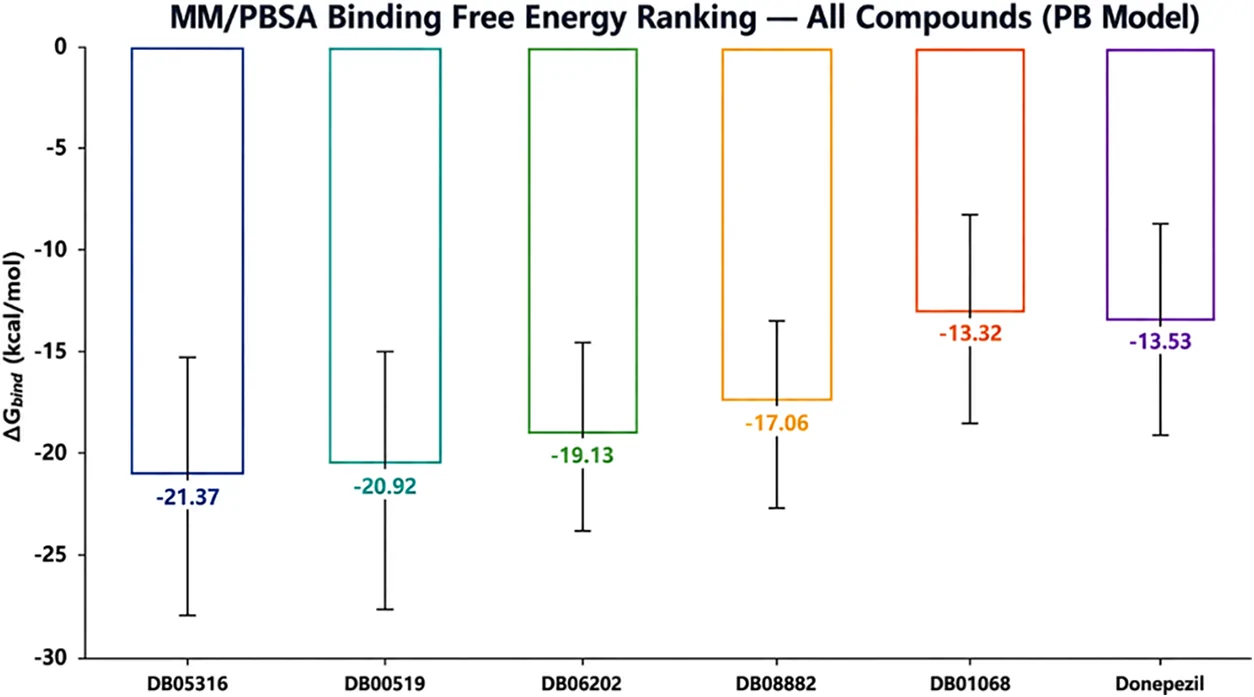
MM/PBSA energy profile of screened drugs in comparison of donepezil.

#### Thermodynamic Binding Profiles and Per-Residue Energy Contributions

Across all systems, the MM/PBSA analyses provide a coherent thermodynamic framework describing ligand recognition and stabilization within the binding pocket. DB00519 exhibits a stable, high-affinity profile, with an ensemble-averaged Δ*G*_(bind)_ of −20.92 ± 5.37 kcal/mol. Its interaction is dominated by hydrophobic packing and shape complementarity, reflected in strong van der Waals contributions (Δ*E*_(vdW)_ = −36.51 kcal/mol) and favorable electrostatics (Δ*E*_(eel)_ = −13.24 kcal/mol), which collectively offset the polar desolvation cost (Δ*G*_(pb)_ = 33.31 kcal/mol). Residues 152, 210, and 259 emerge as key stabilizing anchors. DB01068 maintains a stable but weaker binding configuration (Δ*G*_(bind)_ = −13.32 ± 3.00 kcal/mol). Its affinity is driven mainly by non-polar interactions (Δ*E*_(vdW)_ = −21.69 kcal/mol; Δ*E*_(nonpolar)_ = −2.46 kcal/mol), with modest electrostatic support (Δ*E*_(eel)_ = −4.38 kcal/mol). A substantial polar desolvation penalty (Δ*G*_(pb)_ = 15.21 kcal/mol) limits overall stabilization. Residue 212 contributes the strongest favorable interactions, whereas residue 221 imposes a local desolvation barrier.

DB05316 displays the most favorable thermodynamic signature, with a mean Δ*G*_(bind)_ of −21.37 ± 5.28 kcal/mol and a deep minimum in replicate 2 (−27.13 ± 6.78 kcal/mol). Its exceptional affinity arises from pronounced non-polar forces (Δ*E*_(vdW)_ = −42.25 kcal/mol) and a strongly favorable gas-phase component (Δ*G*_(gas)_ = −53.46 kcal/mol). Although penalized by polar desolvation (Δ*G*_(pb)_ = 36.48 kcal/mol), the compound remains strongly stabilized through coordinated interactions at residues 152, 210, and 256. DB06202 also demonstrates robust binding (Δ*G*_(bind)_ = −19.13 ± 3.50 kcal/mol), with replicate 1 showing enhanced affinity of (−22.80 ± 3.47 kcal/mol). Its stabilization is driven by non-polar contacts (Δ*E*_(vdW)_ = −32.88 kcal/mol; Δ*E*_(nonpolar)_ = −3.98 kcal/mol) and moderate electrostatics (Δ*E*_(eel)_ = −7.49 kcal/mol), partially offset by polar desolvation (Δ*G*_(pb)_ = 25.21 kcal/mol). Residues 152, 210, and 309 form the principal interaction network, though residue 210 also contributes an unfavorable desolvation component. DB08882 exhibits a stable yet comparatively weak binding profile (Δ*G*_(bind)_ = −13.53 ± 5.15 kcal/mol). Despite strong gas-phase stabilization (Δ*E*_(vdW)_ = −39.90 kcal/mol; Δ*E*_(eel)_ = −21.11 kcal/mol), its affinity is severely diminished by an exceptionally high polar desolvation penalty (Δ*G*_(pb)_ = 52.32 kcal/mol), yielding a large positive solvation energy (Δ*G*_(solv)_ = 47.49 kcal/mol). Residues 152, 210, and 309 contribute stabilizing interactions, but residue 210 imposes a strong unfavorable polar desolvation barrier. Donepezil, the reference drug, shows a consistent binding profile (Δ*G*_(bind)_ = −17.06 ± 5.19 kcal/mol), with replicate 3 yielding the most stable value (−17.99 ± 4.20 kcal/mol). Its affinity is driven by non-polar forces (Δ*E*_(vdW)_ = −33.65 kcal/mol; Δ*E*_(nonpolar)_ = −4.07 kcal/mol) and supported by electrostatics (Δ*E*_(eel)_ = −13.70 kcal/mol) but offset by polar desolvation (Δ*G*_(pb)_ = 34.37 kcal/mol). Residue 152 dominates the interaction landscape, with residue 210 acting as a secondary stabilizer. A comparative analysis reveals a clear thermodynamic hierarchy: DB05316 > DB00519 > DB06202 > Donepezil > DB08882 > DB01068. The top-ranking compounds benefit from enhanced shape complementarity and extensive non-polar contact networks, enabling multi-point anchoring across residues 152, 210, and 256/309. In contrast, Donepezil relies heavily on a single hotspot at residue 152, rendering it more susceptible to local perturbations. The broader interaction footprint of the lead scaffolds confers superior energetic stability and highlights their potential as more resilient therapeutic candidates (Figure 26).

**Figure 26:**
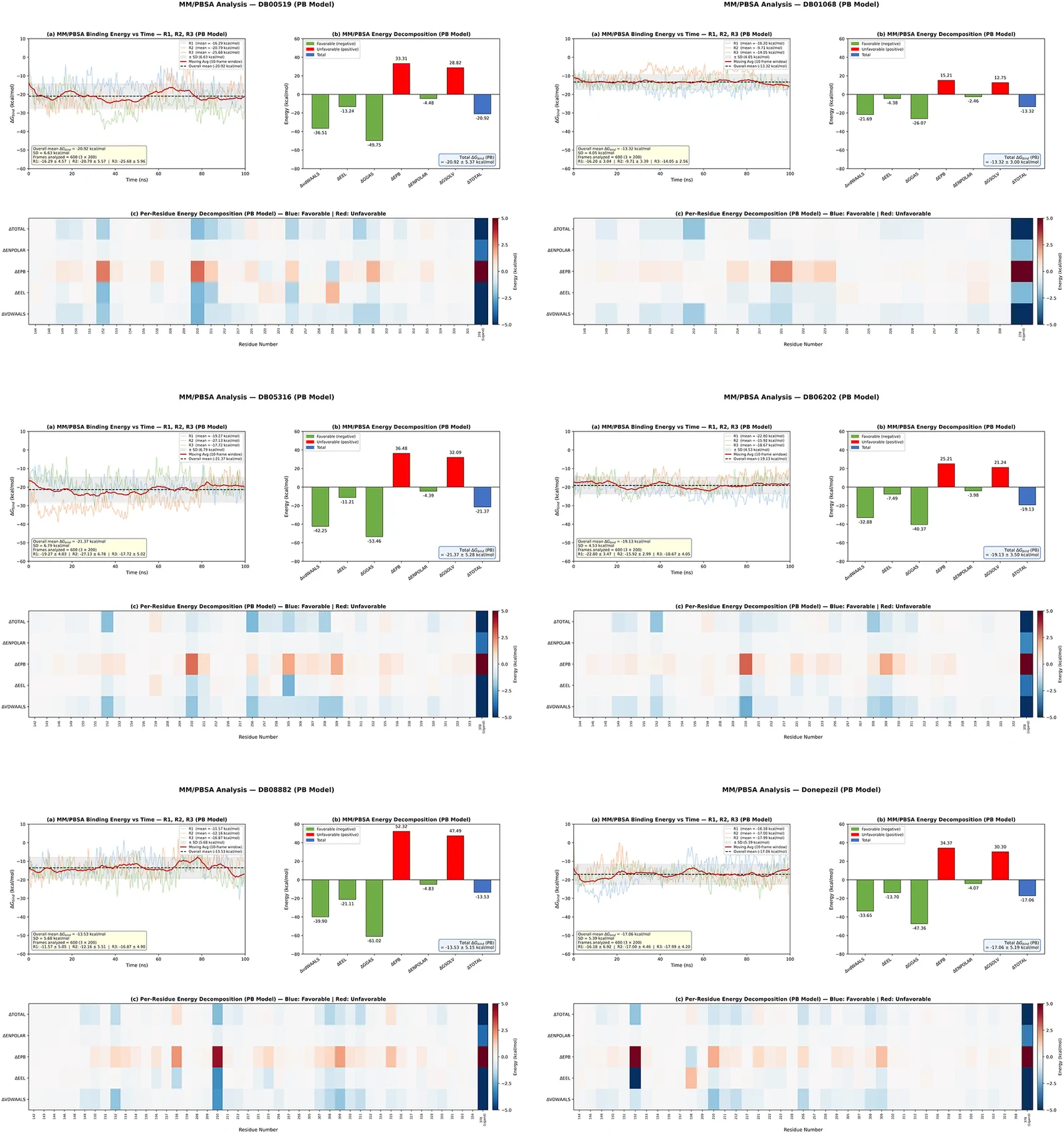
MM/PBSA binding-energy landscapes (a–c) summarize the thermodynamic stability, energetic decomposition, and residue-level interaction profiles of all six ligand-protein complexes, highlighting dominant non-polar contributions and key anchoring residues.

## Conclusion

In this study, we developed an integrated ligand-based virtual screening framework to identify potential CASP4 inhibitors from the DrugBank database, combining docking-score prioritization, SMILES-derived ChemBERTa embeddings, and key physicochemical descriptors. The Random Forest classifier trained on the hybrid ChemBERTa physicochemical feature set demonstrated strong predictive performance, achieving ∼95% accuracy (ROC–AUC = 0.73) and a ∼3.5-fold early enrichment of active compounds, while a companion Random Forest regressor trained on experimental pIC_50_ values of active compounds was used to rank candidates by predicted potency. Docking results showed that DB01068 (−9.75 kcal/mol), DB09477 (−8.68 kcal/mol), DB05316 (−8.51 kcal/mol), DB08882 (−8.35 kcal/mol), and DB06202 (−8.0 kcal/mol) exhibited comparable better binding energy values compared to standard donepezil (–7.9 kcal/mol). Moreover, MD simulations combined with MM/PBSA free-energy calculations further distinguished DB05316 (Δ*G*_bind_ −21.37) and DB00519 (Δ*G*_bind_ −20.92 kcal/mol) as the most energetically stable ligand across replicates, giving it a clear performance advantage over the other compounds. Taken together, our findings demonstrate a robust and efficient computational framework for identifying repositioned CASP4 inhibitors and provide a strong foundation for future biochemical validation and therapeutic development for AD.

## Supplementary Material







## Data Availability

All data, input files, docking results, and analysis scripts required to reproduce this study are included in the Supporting Information. Additional source code, computational workflows, and processed datasets are openly accessible at our GitHub repository: https://github.com/mubashirhassangcul/Caspase4-inhibitor-screening.

## Notes

Filename; score; and cluster id (CSV)
Drugbank id; chembl id; smiles; canonical smiles; token count; pIC_50_, and active (CSV)
Structural features relevant to CASP4 inhibitor modeling; protein preparation workflow, and citation (PDF)
