# Supplementary material for: AI Accelerated Chemical Screening Integrates ChemBERTa to Identify Repositionable CASP4 Inhibitors via MD Simulations and MM/PBSA Analysis
Source: J Chem Inf Model. 2026 Sep 14;66(19):12740–71. doi: 10.1021/acs.jcim.6c02284 (PMC13663865; doi:10.1021/acs.jcim.6c02284)
Supplement: Supplementary file 1 [file ci6c02284_si_003.pdf]

SUPPORTING INFORMATION

AI accelerated chemical screening integrates ChemBERTa to identify repositionable CASP4 inhibitors via MD simulations and MM/PBSA analysis

Mubashir Hassan*[1], Sidra Ghayour Bhatti[2], Muhammad Yasir[3], Wanjoo Chun[3], Andrzej Kloczkowski[1,4]*

[1]The Steve and Cindy Rasmussen Institute for Genomic Medicine at Nationwide Children's Hospital, Columbus, Ohio, USA, 43205. Email: mubashirhassan_gcul@yahoo.com; Mubasher.Hassan@nationwidechildrens.org. ORCID: https://orcid.org/0000-0003-2532-1866

[2]The Ohio State University, Columbus, Ohio, USA, 43210. Email: bhatti.39@osu.edu. ORCID: https://orcid.org/0000-0003-4094-5984.

[3]Department of Pharmacology, Kangwon National University School of Medicine, Chuncheon, Republic of Korea, 24341. Email: Yasir.khokhar1999@gmail.com. ORCID: https://orcid.org/0009-0001-7248-8741.

[4]Department of Pharmacology, Kangwon National University School of Medicine, Chuncheon, Republic of Korea, 24341. Email: wchun@kangwon.ac.kr. ORCID: https://orcid.org/0000-0003-1984-3545

MH and SGB contributed equally to this work as co-first authors

Correspondence

Andrzej Kloczkowski (Ph.D)

Email: Kloczkowski.1@osu.edu; Andrzej.Kloczkowski@nationwidechildrens.org

Mubashir Hassan (Ph.D)

Email: mubashirhassan_gcul@yahoo.com; Mubasher.Hassan@nationwidechildrens.org

S1.1 Rationale for Selecting PDB ID 6NRY

PDB ID 6NRY was chosen as the receptor structure for molecular docking because it provides a high‑quality, experimentally resolved conformation of human caspase‑4 (CASP4). The structure contains a complete catalytic domain with clearly resolved active‑site residues, including the catalytic cysteine, which is essential for modeling ligand interactions. The crystallographic resolution and absence of major missing loops or disordered regions make 6NRY a reliable template for structure‑based inhibitor screening.

Experimental Data

- **Method:** X-RAY DIFFRACTION
- **Resolution:** 2.18 Å
- **R-Value Free:** 0.237 (Depositor), 0.240 (DCC)
- **R-Value Work:** 0.195 (Depositor), 0.198 (DCC)
- **R-Value Observed:** 0.199 (Depositor)

Unit Cell:

Length (Å)	Angle (˚)
a = 40.687	α = 90
b = 163.383	β = 90
c = 103.661	γ = 90

S1.2 Structural Features Relevant to CASP4 Inhibitor Modeling

The 6NRY structure exhibits several characteristics that support its suitability for docking studies:

A well‑defined active site with stable backbone and side‑chain orientations.

Properly resolved Cys258 and surrounding residues that govern ligand recognition.

A clearly identifiable substrate‑binding groove, enabling consistent definition of the docking pocket.

No significant steric clashes or unresolved electron density near the catalytic region.

These features ensure that ligand placement and scoring are not compromised by structural artifacts.

S1.3 Protein Preparation Workflow

Prior to docking, the 6NRY structure was processed using a standardized receptor-preparation protocol: Removal of crystallographic water molecules not involved in stable hydrogen-bond networks. Addition of hydrogen atoms according to the selected force-field protonation scheme. Assignment of protonation states for ionizable residues at physiological pH. Optimization of Asn, Gln, and His side-chain orientations. Brief energy minimization to relieve steric clashes and stabilize the active-site geometry.

S1.4 Relevance to CASP4 Computational Studies

The 6NRY structure has been used in prior computational analyses of CASP4 and represents a biologically meaningful conformation for evaluating potential inhibitors. Its structural completeness and active-site clarity make it an appropriate reference for docking, molecular dynamics simulations, and MM/PBSA free-energy calculations.

S1.5 Citation

Protein structure source:

PDB ID: 6NRY Human Caspase-4.

Deposited: 2019-01-24 Released: 2020-07-29

Deposition Author(s): Yang, J., Liu, Z., Shi, W., Xiao, T.S.

Funding Organization(s): National Institutes of Health/National Human Genome Research Institute (NIH/NHGRI)

https://files.rcsb.org/validation/view/6nry_full_validation.pdf
